# Carriage of λ Latent Virus Is Costly for Its Bacterial Host due to Frequent Reactivation in Monoxenic Mouse Intestine

**DOI:** 10.1371/journal.pgen.1005861

**Published:** 2016-02-12

**Authors:** Marianne De Paepe, Laurent Tournier, Elisabeth Moncaut, Olivier Son, Philippe Langella, Marie-Agnès Petit

**Affiliations:** 1 Micalis Institute, INRA, AgroParisTech, Université Paris-Saclay, Jouy-en-Josas, France; 2 MaIAGE, INRA, Université Paris-Saclay, Jouy-en-Josas, France; Uppsala University, SWEDEN

## Abstract

Temperate phages, the bacterial viruses able to enter in a dormant prophage state in bacterial genomes, are present in the majority of bacterial strains for which the genome sequence is available. Although these prophages are generally considered to increase their hosts’ fitness by bringing beneficial genes, studies demonstrating such effects in ecologically relevant environments are relatively limited to few bacterial species. Here, we investigated the impact of prophage carriage in the gastrointestinal tract of monoxenic mice. Combined with mathematical modelling, these experimental results provided a quantitative estimation of key parameters governing phage-bacteria interactions within this model ecosystem. We used wild-type and mutant strains of the best known host/phage pair, *Escherichia coli* and phage λ. Unexpectedly, λ prophage caused a significant fitness cost for its carrier, due to an induction rate 50-fold higher than *in vitro*, with 1 to 2% of the prophage being induced. However, when prophage carriers were in competition with isogenic phage susceptible bacteria, the prophage indirectly benefited its carrier by killing competitors: infection of susceptible bacteria led to phage lytic development in about 80% of cases. The remaining infected bacteria were lysogenized, resulting overall in the rapid lysogenization of the susceptible lineage. Moreover, our setup enabled to demonstrate that rare events of phage gene capture by homologous recombination occurred in the intestine of monoxenic mice. To our knowledge, this study constitutes the first quantitative characterization of temperate phage-bacteria interactions in a simplified gut environment. The high prophage induction rate detected reveals DNA damage-mediated SOS response in monoxenic mouse intestine. We propose that the mammalian gut, the most densely populated bacterial ecosystem on earth, might foster bacterial evolution through high temperate phage activity.

## Author Summary

Dormant bacterial viruses, or prophages, are found in the genomes of almost all bacteria, but their impact on bacterial host fitness is largely unknown. Through experiments in mice, supported by a mathematical model, we quantified the activity of *Escherichia coli* prophage λ in monoxenic mouse gut, as well as its impact on its carrier bacteria. λ carriage negatively impacted its hosts due to frequent reactivation, but indirectly benefited its host by killing susceptible bacterial competitors. The high prophage activity unraveled in this study reflects a constant rate of SOS response, resulting from DNA damage in monoxenic mouse intestine. Our results should motivate researchers to take the presence of prophages into account when studying the action of specific bacteria in the gastrointestinal tract of mammals.

## Introduction

Bacterial viruses, called bacteriophages or phages, are present in all bacterial communities and have profound impact on bacteria either by killing them or by mediating horizontal gene transfer through lysogeny. Lysogeny refers to the ability of temperate phages, as opposed to virulent ones, to repress their lytic multiplication after infection and stably segregate with the bacteria. In most cases, the repressed phage, or prophage, is integrated into the bacterial chromosome, but it can also replicate as an extrachromosomal element in the bacterium. Nearly all bacterial genomes contain one or multiple prophages, which can constitute up to 14% of the genome for *Escherichia coli* strains [1]. Active prophages can be induced, i.e. switch back to lytic multiplication in response to a signal such as DNA damage and subsequent SOS response (reviewed in [2]). Induction rates are usually too low to result in a cost to their host, and prophages were generally found to have positive impacts on lysogenic bacteria [3–6]. The benefits of lysogeny can result from three distinct mechanisms: (i) lysogenic conversion, by which phages bring useful bacterial accessory traits [4,7]; (ii) immunity, i.e. protection against other phages, as the prophage protects its carrier bacterium against the same, and sometimes other, phages [8]; and (iii) allelopathy, by releasing infectious virions that are able to kill susceptible bacterial competitors. While induction results in the death of the lysogen, it can provide a competitive advantage for the remaining lysogenic population. A large number of major bacterial toxins, such as the diphtheria, Panton-Valentine, cholera, Shiga- or scarlatin toxins are encoded on temperate phage genomes (reviewed in [7]). However, pathogenicity does not always increase bacterial fitness in a human host, suggesting that some pathogenic traits can be coincidental (reviewed in [9]). To our knowledge, except for *Staphylococcus aureus*, only a small proportion of prophages were demonstrated to carry beneficial traits for their bacterial host, such as improvement of the colonization of body surfaces—like intestine [10,11], nasopharynx [12], or skin [13]—or resistance to protozoa grazing [14,15]. The allelopathic character of temperate phages has been demonstrated by *in vitro* experiments and mathematical modelling [16,17], but also recently during insect infection [18]. However, very few data exist concerning the impact of prophages on the fitness of their hosts in the most densely populated bacterial ecosystem, the intestine of mammals. Metagenomic studies have shown that gut bacteria harbor many temperate phages [19], but whether carrying a prophage is generally costly or advantageous for its host has been rarely investigated in the intestinal environment [20,21]. A well documented case of beneficial interaction is the filamentous temperate phage of *Vibrio cholerae* VPIΦ, which encodes factors essential for bacterial adherence and intestine colonization [10,11]. *E*. *coli* prophages carrying Shiga toxin *stx* genes are known to be active in the intestine, but their excision and lysogenization rates were not quantified [22,23]. Another study demonstrated that a prophage of an *Enteroccocus faecalis* strain provided a 1.5-fold growth advantage after 24 hours of mouse gut colonization [24], but the mechanisms involved were not entirely explored, nor the impact of prophage presence after the first day of colonization.

The costs or benefits of lysogeny in the gastrointestinal tract cannot be inferred from *in vitro* studies, since the parameters that rule phage-bacteria interactions vary greatly with the environment, bacterial physiology and medium structure. For example, the lysogenization rate of phage λ, i.e. the proportion of infected *E*. *coli* bacteria that are lysogenized upon infection, varies from 10^−3^ when infecting cells in optimal growth conditions, to 0.5 when infecting starved cells [25]. This rate also varies with temperature and multiplicity of infection [26]. Three other main interaction parameters can be distinguished: (i) the induction rate, (ii) the adsorption rate onto the bacterial host, i.e. affinity of the phage for its receptors, a parameter that greatly depends on ionic conditions [27], and (iii) the multiplication rate within the host. Up to now, none of these parameters has been determined for a temperate phage in the gut environment. Yet, characterizing temperate phage activity is essential to estimate their impact on lysogenic bacteria, and to evaluate the extent of the horizontal gene transfer they mediate in this environment. This point is of paramount importance because temperate phages are major actors of bacterial genome evolution, and as such they participate to the emergence of new pathogenic strains. Moreover they are suspected to be important disseminators of antibiotic resistance genes [28].

The extreme complexity of the gut microbiota prevents any exhaustive characterization of all the virus-host systems it hosts. It is thus necessary to first characterize specific virus–host systems in a controlled microbiota to bridge the existing gap between *in vitro* studies and the functional characterization of natural gut microbial communities. We used monoxenic mice, i.e. mice associated with a single bacterial species, to perform competition experiments between two isogenic *E*. *coli* strains, one carrying the λ prophage and the other devoid of it. These experiments, supported by a mathematical model consisting of five ordinary differential equations, allowed disentangling the different components of the impact of the prophage on bacterial reproductive fitness. We obtained quantitative estimations of the main parameters driving phage-bacteria interactions in monoxenic mouse intestine. Moreover, we demonstrate that efficient phage spreading enabled rare events of phage-mediated gene capture by homologous recombination, and transmission to new bacteria.

## Results

### Monoxenic mouse gut environment allows for rapid phage multiplication

**Fig 1:**
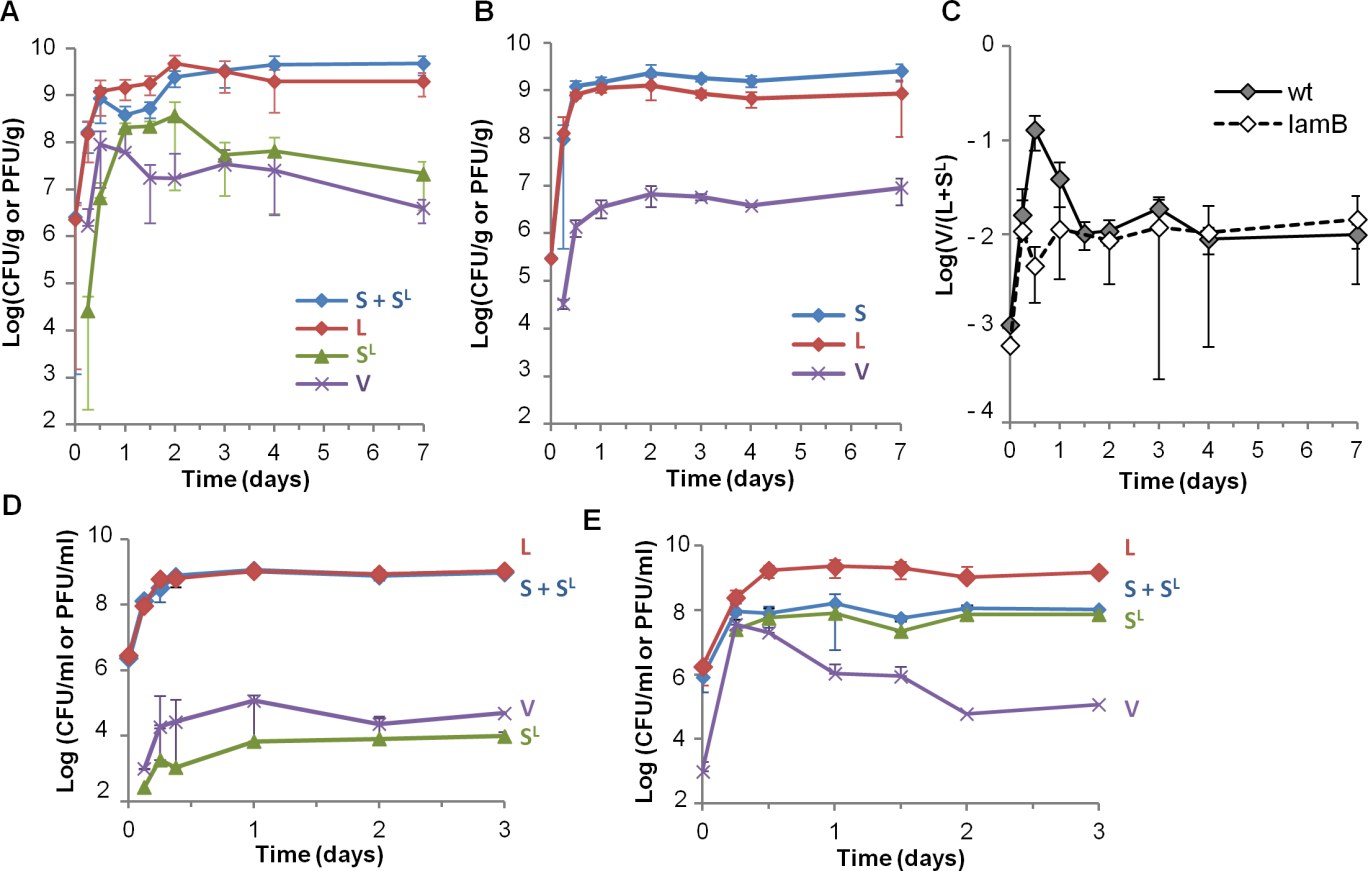
Rapid phage propagation occurs during the first day of colonization. A) Temporal evolution of bacterial lineages S, L and S^L^ and free phage V densities in mouse feces: L bacteria (red), bacteria from the susceptible lineage (S + S^L^, blue), S^L^ bacteria (green), free phage (purple, V). Means +/- standard deviation from 8 mice in 2 independent experiments are indicated. B) Temporal evolution of S, L and free phage V densities in feces of mice colonized with *lamB* derivatives of bacterial strains. Means +/- standard deviations from 6 mice are indicated. C) Temporal dynamics of the free phage (V) over total lysogen (L + S^L^) ratio in mouse feces. Mice were colonized either with the two strains of panels A (wt, full line), or with their *lamB* derivatives described in panel B (dashed line). Means +/- standard errors from 8 and 6 mice for the wt and *lamB* strains respectively are indicated. D) Temporal dynamics of bacterial lineages S, L, S^L^ and free phage V subjected to serial transfers in LB flasks. Phage propagation is very limited, as observed by the low density of S^L^ bacteria and free phage V. E) Same populations in LB supplemented with 5 mM MgSO_4_. Means +/- standard deviations of ratios on 4 independent cultures are indicated.

To characterize phage-bacteria interactions in the mouse digestive tract, we colonized germ-free mice with two isogenic *E*. *coli* MG1655 strains, except for antibiotic resistance markers and the presence of the λ prophage (λ*ble* phage confering phleomycin resistance to the lysogen). Populations of free phage (V), bacteria from the lysogenic lineage (L), from the susceptible lineage (S) and newly lysogenized by λ (S^L^) were quantified in mouse feces for one week, based on their differential antibiotic resistance levels. During the first day of colonization, phage propagation, *via* free phage production and lysogenization of the susceptible bacteria, was highly efficient: after 24 hours of colonization, an average 73% of the bacteria from the initially susceptible lineage were either killed or lysogenized, and a transient increase in free phage had occurred (Fig 1A). To determine what fraction of free phage was produced by multiplication on susceptible bacteria, as opposed to free phage produced by spontaneous induction in lysogens, the same experiment was repeated with *lamB* derivatives of the two strains, devoid of the phage receptor and resistant to phage infection (Fig 1B). In such conditions all free phages result from the spontaneous induction of prophages in lysogenic bacteria. At the peak of free phage production, the free phage over lysogen ratio was 20-fold lower than in the experiment with wt strains (Fig 1C), indicating that the transient increase of free phage observed with these strains resulted from multiplication on susceptible bacteria, and not from a transient increase in induction rate.

By comparison, when the same S and L strains were co-cultured *in vitro*, in standard rich LB medium, phage propagation was almost undetectable (Fig 1D), in line with previously published results [29]. This absence of propagation was due to low Mg^2+^ concentration in LB, drastically limiting λ adsorption ([27] and Fig 1D and 1E). Addition of maltose did not improve phage propagation, suggesting that LamB expression in LB is sufficient for phage infection [30].

### Phage propagation is impeded by *malT* mutations after 1.5 days of colonization

**Fig 2:**
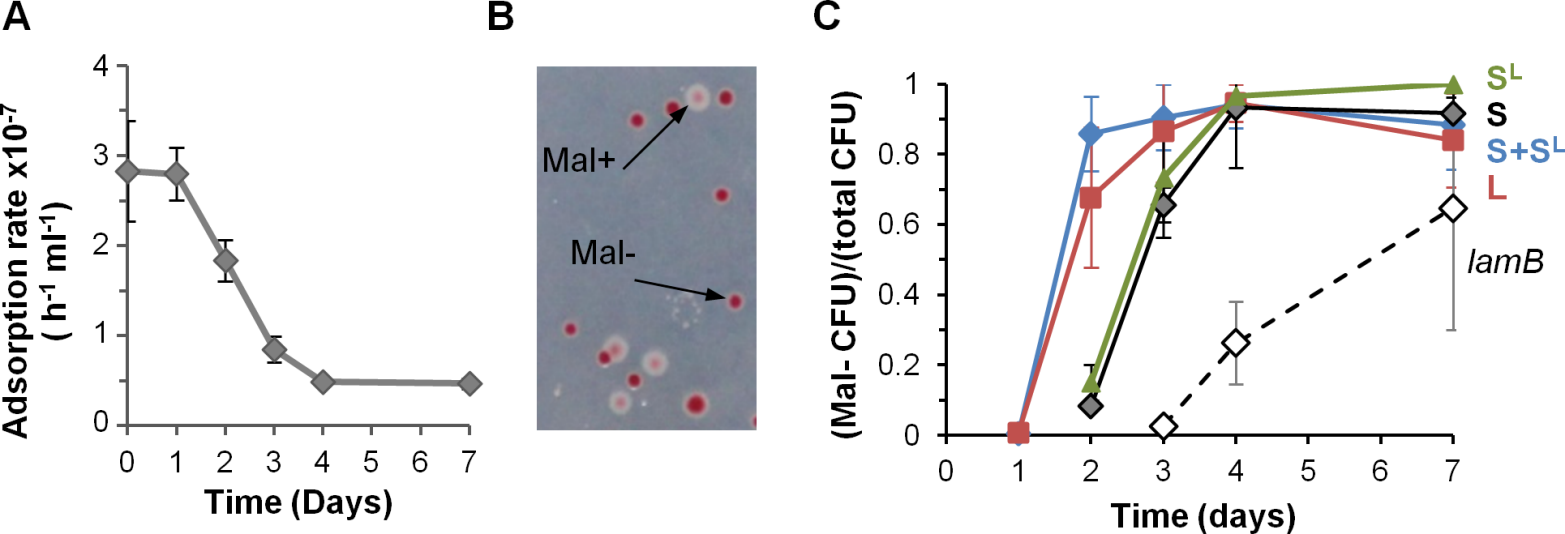
Resistant mutants invade independently of phage presence. A) Evolution of the phage adsorption rate on bacteria from mouse feces. On day 1 the adsorption rate was close to its maximal theoretical value. Means +/- standard errors from three mice are indicated. B) Detection and enumeration of Mal^-^ colonies on tetrazolium maltose plates after 2 days of colonization: white, wild-type Mal^+^ colonies; red, Mal^-^ colonies. Mal- colonies have mutations in *malT*, the positive regulator of the maltose operon, and do not express the phage receptor LamB. C) Evolution of the proportion of Mal- bacteria with time in the S and L lineages. In addition to the results of the co-colonization experiments, the proportion of Mal^-^ bacteria during two independent monocolonization experiments are indicated: black line, monocolonization with the S strain; dotted line, monocolonization with a *lamB* strain. Means +/- standard deviations of four mice in each experiment are indicated.

In mice, phage propagation stopped before the complete lysogenization of the S lineage. To test whether this resulted from changes in gut bacteria impairing infection, mice were monocolonized with the susceptible strain S only, and bacteria from feces were tested for affinity to λ (Fig 2A). After one day, λ adsorption rate on bacteria from mouse feces was measured at 3.10^−7^ ml h^-1^, which is similar to the value measured at day 0 *in vitro*. Therefore the phage receptor LamB is highly expressed in the mouse gut, and favorable ionic conditions allow for efficient binding. Later on however, the adsorption rate diminished continuously, suggesting a decrease in LamB expression (Fig 2A). To investigate this phenomenon further, we determined the susceptibility to λ of S clones isolated from mouse feces two days after colonization. Nine out of the twelve clones tested turned out to be genetically resistant to λ, and were moreover unable to use maltose, as revealed by their inability to grow on minimal medium containing maltose as the unique energy source. In subsequent colonization experiments, we quantified the increase in maltose-deficient bacteria (Mal^-^) by using maltose agar plates containing a tetrazolium dye that turned red in Mal^-^ colonies (Fig 2B). Mal^-^ bacteria were selected in the S and L lineages (Fig 2C). A similar rise in Mal^-^ bacteria occurred in mice monocolonized with the phage-free strain S, demonstrating unambiguously that their selection is not caused by λ (Fig 2C).

Mal^-^ and λ resistance phenotypes, as well as previously published results [31], guided our identification of mutations in the *malT* gene. MalT is the transcriptional activator of the maltose regulon. It notably controls expression of the λ receptor LamB. All six resistant clones studied carried a mutation in *malT*, among which three led to a truncated protein (S1 Fig), which explains that the selected mutations prevent phage infection. The reason for the selection of these mutants might be linked to the LamB-induced envelope stress associated with osmoregulation [30] since bacteria in the gastrointestinal lumen are continuously exposed to osmotic stress (reviewed in [32]). They are specific to monoxenic mice, as *malT* mutations are not selected for in the MG1655 *E*. *coli* strain when colonizing mice with a conventional microbiota [33].

### Mathematical modelling of phage-bacteria interactions in monoxenic mouse gut

**Fig 3:**
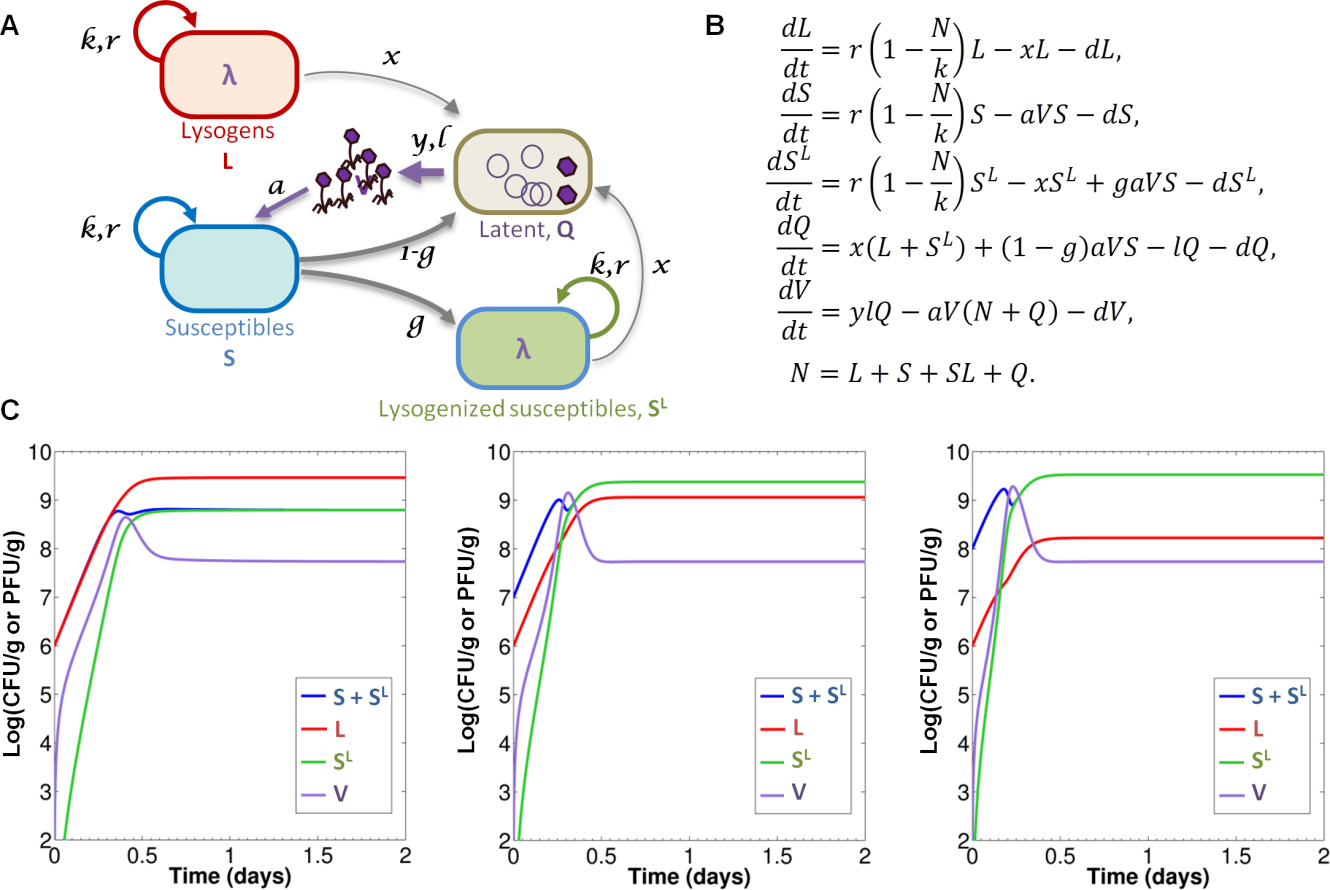
Quantitative mathematical modelling of temperate phage-bacteria interactions. A) Diagram representing the interactions between the different populations. S and L lineages grow with a common maximal growth rate *r* and carrying capacity *k*. Lysogenic populations (L and S^L^) switch to lysis at a rate *x* entering latent population Q. Phage binding on bacteria is determined by the adsorption constant *a*. Infection of susceptible leads either to lysogenization with probability *g*, or to lytic cycle with probability 1-*g*. Latency rate *l* and burst size *y* determine the production of free phage V. The dilution term *d* accounts for the mouse gastrointestinal flux. B) System of differential equations governing the dynamics of the five populations (variable N denotes total population L+S+S^L^). C) Numerical simulations of the model with estimated values of Table 1: the lines represent the temporal evolution of population densities S+S^L^ (blue), L (red), S^L^ (green) and V (purple), with different initial conditions: L_0_ = 1x10^6^ cfu/g and, from left to right, S_0_ = L_0_, S_0_ = 10×L_0_, S_0_ = 100×L_0_.

The rise of *malT* mutants was nevertheless sufficiently delayed to permit observation phage infection of the majority of S bacteria during the first two days. In order to provide quantitative estimations of the parameters governing phage-bacteria interactions, we developed a mathematical model representing the dynamics of the different microbial populations in this model ecosystem (Fig 3). The model is based on the one in [17] and adapted to take into account our experimental settings. It consists of five coupled differential equations, representing time evolution of five population densities: S (susceptibles), L (lysogens), S^L^ (newly-lysogenized susceptibles), V (free phage), as well as latent bacteria Q in which the phage undergoes lytic multiplication. Invasion of *malT* mutants is not included in the model. S1 Text gives a detailed description of the main modeling assumptions behind its construction, as well as a mathematical analysis of its dynamical behavior.

A careful examination of the effect of the eight model parameters onto the dynamics enabled the quantitative estimation of six of them from our experimental datasets (Table 1). With these estimated values, numerical simulations of the model (Fig 3C) are in good agreement with experimental observations on the first two days, before invasion of *malT* mutants, suggesting it captures most of the relevant information contained in our data. The main discrepancy observed is in the initial velocity of the temporal evolutions, faster in the model than in experimental data. This might result either from incorrect estimation of some parameters, or from the neglect of a phenomenon not taken into account in the model, such as the binding of free phage on some intestinal components. Such binding would result in a “loss” of phage that would slow the dynamics, as exemplified by the effect of reduced burst size (S2 Fig).

**Table 1:** Estimated values of the model’s parameters.

	Description	Value	95% Conf. interv.	Comments
*d*	Intestinal dilution rate	0.25 h^-1^	-	Global parameter, fixed.
*r*	Maximal growth rate	1.1 h^-1^	1.1 ; 1.18	Bacterial growth parameters, estimated from single-species experiments.
*k*	Carrying capacity	4.6 x 10^9^ cfu g^-1^	4.3 10^9^ ; 5.0 10^9^	
*x*	Induction rate	0.016 h^-1^	6.3 10^−3^ ; 3.6 10^−2^	Estimated from densities of L, S, Q in lamB derivative experiments.
*l*	Latency rate	0.8 h^-1^	0; 1.5	
*y*	Burst size	12.1	-	Calculated from *in vitro* experiments.
*a*	Adsorption constant	2.6 x 10^−9^ g h^-1^	-	Estimated from L-S competition experiments.
*g*	Lysogenization rate	0.19	-	

### High lysogenization limits the final gain of the original prophage carrier strain

**Fig 4:**
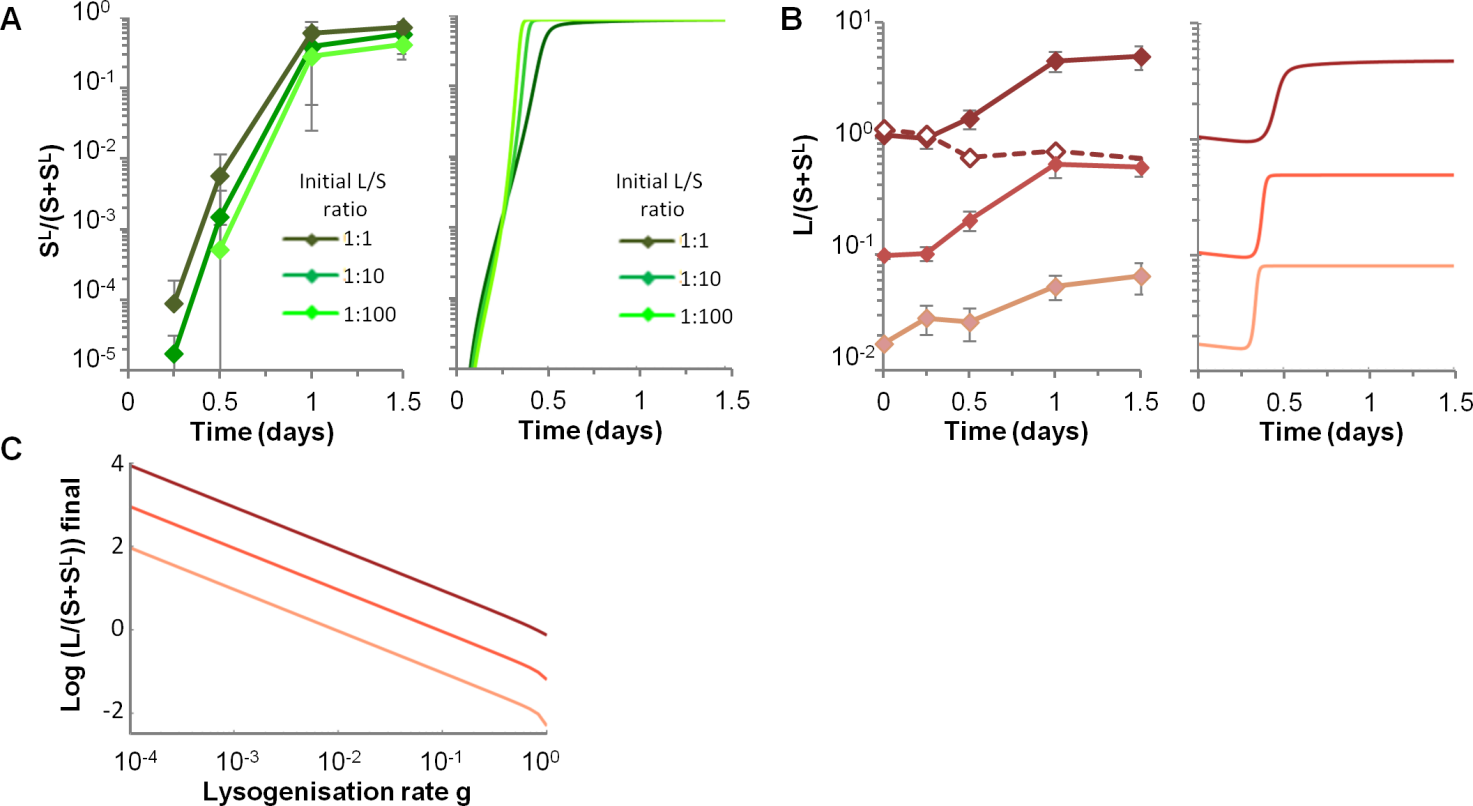
High lysogenization limits the final gain of the original prophage carrier strain. A) Temporal dynamics of the proportion of newly lysogenized bacteria (S^L^/(S+S^L^)) for three different initial L/S ratios, in the experimental data (left panel) and with numerical simulations of the model (right panel). Means +/- standard deviations from 8, 13 and 4 mice are indicated for L/S initial ratios 1:1, 1:10 and 1:100 respectively. In simulations, initial conditions for L and S densities are those of respective data. B) Temporal dynamics of the ratio of L over S lineages (L/(S+S^L^)), depending on the three initial L/S ratios (1:1, 1:10 and 1:100), in the experimental data (left panel) and in the simulation (right panel). Means +/- standard deviations from 8, 13 and 4 mice are indicated for L/S initial ratios 1:1, 1:10 and 1:100 respectively. In simulations, initial L and S densities are the mean of respective data. C) Impact of the lysogenization rate g on the final L over S lineages ratio, as predicted by the mathematical model.

Upon infection of a susceptible bacterium, λ goes to lysogenization with a high probability around 19%, leading to a very rapid rise of S^L^ bacteria both in data and in numerical simulations (Fig 4A). The remaining infected susceptibles were lysed, resulting in an increase of the L lineage relative to the S one, independently of the initial L/S ratios (Fig 4B). *LamB* deletion abolished the competitive advantage of the L lineage (Fig 4B, dotted line), confirming that the advantage of lysogens only stemmed from the lysis of susceptible competitors, and not from the presence of putative bacterial fitness genes in the λ genome that would improve growth in mice. However, the gain of the L lineage over the S one is limited by the lysogenization of susceptible, independently from the rise of λ resistant mutants. Its final value, as predicted by the model, seems to be directly proportional to the inverse of *g* at population equilibrium (Fig 4C). Interestingly, other parameters governing phage-bacteria interaction have very modest impact on the final gain of the L lineage (S3 Fig and S1 Text).

### A high prophage induction rate in the gut decreases lysogen fitness

**Fig 5:**
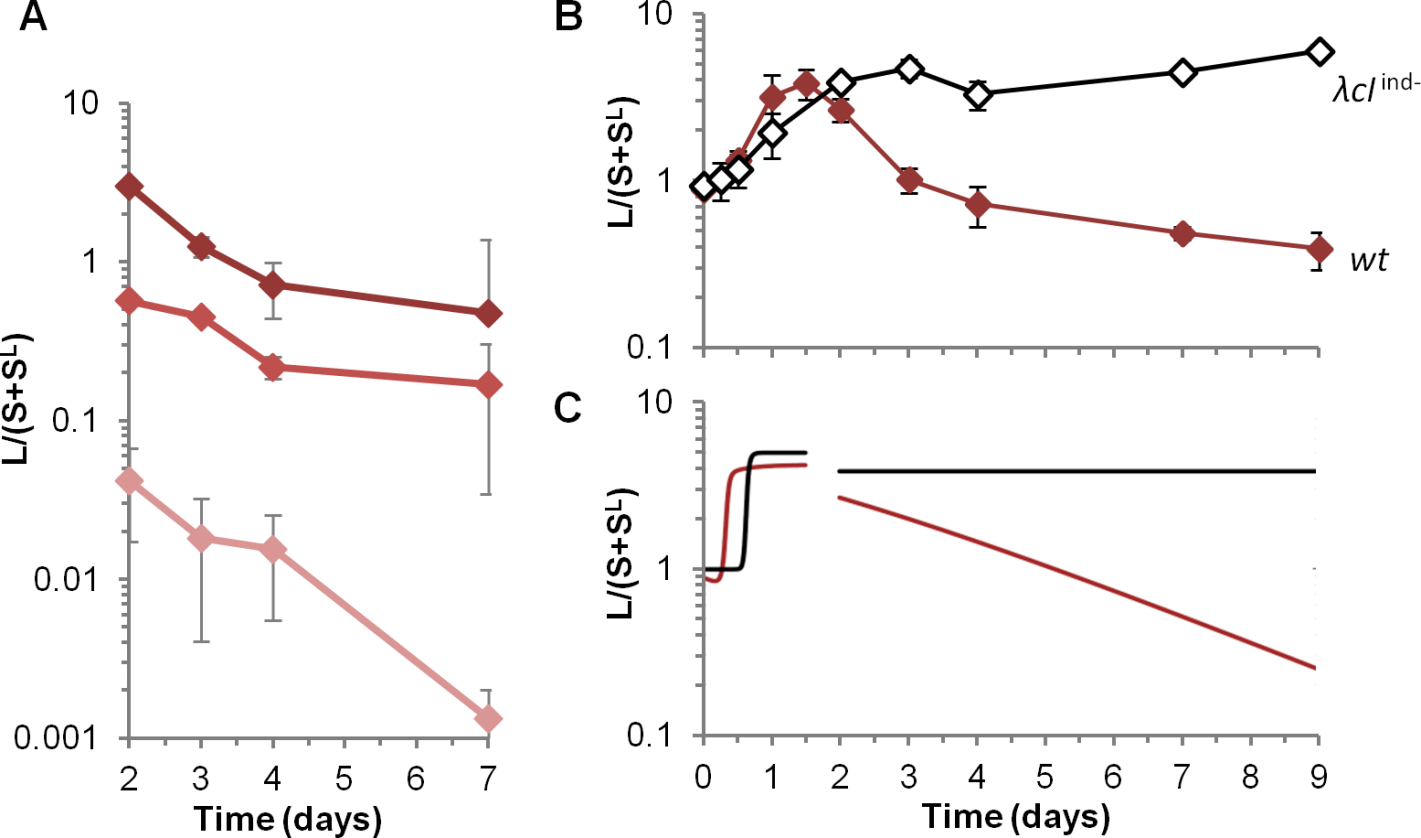
A high *in vivo* induction rate penalizes lysogens in the absence of susceptible bacteria. A) Evolution of the ratio of lysogen over susceptible lineages (L/(S+S^L^)) in mice feces after day 2. Means +/- standard deviations from 8, 13 and 4 mice for initial L/S ratios (1:1, 1:10 and 1:100 respectively) are indicated. B) Evolution over 9 days of L/(S+S^L^) ratio for wild-type λ prophage (brown line) or for λ*cI*^ind-^ deficient for induction (black line). Means +/- standard error of the mean from 7 and 12 mice for λ*cI*^ind-^ and wild-type respectively. C) Two-phase temporal simulation of L/(S+S^L^) ratio, with a switch at 48h to a lamB^-^ version of the model (i.e. with a = 0). The ratio is shown for wild-type phage (red line) or λ*cI*^ind-^ mutant (blue line, with *x* = 2x10^-7^). The initial condition for the first phase is taken from data at time 0, and the initial condition for the second phase (“mutant model”) is taken from experimental data at 48h.

Prophage induction rate in the mouse gastrointestinal tract was estimated to be 1.6%, several orders of magnitude higher than usually assumed. This high induction leads to a slight but systematic decrease of lysogens (L and S^L^ lineages) in mice when bacteria are resistant to infection, either because of *malT* or *lamB* mutations (Figs 1A and 1B and 5A). By contrast, *in vitro*, induction rate is 3 x 10^−4^ (Table 2), and competitions under conditions that did not permit phage infection resulted in a stable proportion over time of lysogenic (L and S^L^ lineages) and non-lysogenic S bacteria (Figs 1D and S3A). In mouse, model-based estimation of the induction rate was derived from latent Q cell counts in mouse colonized with *lamB* strains. Because of the small data set available (one experiment with three mice), the confidence interval is relatively large (0.6%-3.6%, see Table 1 and S1 Text). In order to strengthen the estimation, we also computed the induction rate from the relative fitness of L compared to S *lamB* lineages (Material & Methods). The value found (1.7% ± 0.5%) was very close to that estimated by the model.

**Table 2:** Induction rate per lysogenic bacteria growing on rich medium (LB). The induction rate was measured on ampicillin plates by scoring infective centers, as described in the Materials and Methods section. Since bile salts were shown to cause DNA damage in bacteria [34], and to induce a *Salmonella* prophage [35], we measured their effect on λ induction rate, but no change was detected. Bile salts were added at a final concentration of 0.8%. Mean ± standard deviation of three independent experiments are indicated.

λ genotype	induction rate *in vitro*
**λ wt**	3.4 x 10^−4^ ± 6.8 x 10^−5^
**λ *cI***^***ind-***^	2.4 x 10^−7^ ± 8.9 x 10^−8^
**λ *cI***^ind*^	1.3 x 10^−2^ ± 1.0 x 10^−3^
**λ wt + bile salts**	1.9 x 10^−4^ ± 2.6 x 10^−5^

To examine experimentally the impact of high induction rate, we used a non-inducible λ prophage, λ*cI*^ind-^, which has a mutation in the repressor of the lytic cycle, CI, preventing its RecA activated auto-cleavage upon DNA damage. As expected, in standard *in vitro* conditions, the *cI*^ind-^ mutation decreased the induction rate 1,000-fold (Table 2). In the mouse gastrointestinal tract, the mutation abolished the decrease in proportion of lysogens (Fig 5B), demonstrating unambiguously that high prophage induction explains the disadvantage of lysogens. Moreover, in a *lamB* genetic background, S and λ*cI*^ind-^ lysogenic strains presented no reproductive fitness differences over 9 days (S4B Fig), validating the model hypothesis that in the absence of lysis and induction, the presence of the prophage makes no difference in growth rate. Interestingly, λ*cI*^ind-^ experiments also validated the absence of a rarity threshold to phage multiplication in the mouse gut: since phage amplify on susceptible bacteria, even a very low initial number of phage can lead to killing of a significant part of S lineage (Fig 5B and 5C).

### Transient selection of λ virulent mutants in λ*cI*^ind-^

**Fig 6:**
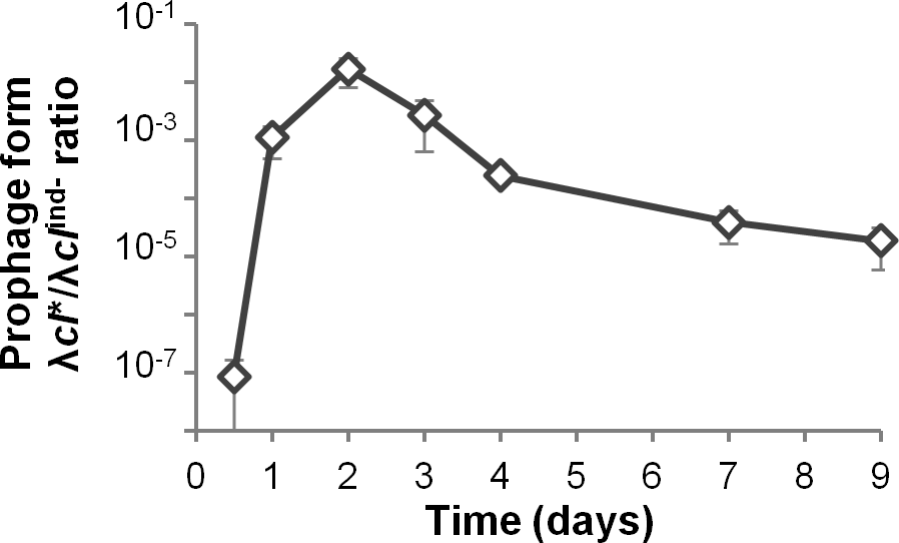
Transient advantage of virulent λcI*phage. Evolution with time of the proportion of bacteria carrying the virulent λcI* prophage (S^L^) on bacteria carrying the λ*c*^ind-^ prophage (L). Means +/- standard error of the mean from 7 mice.

The switch from lysogenic to lytic cycle requires CI autocleavage, catalyzed by RecA nucleofilament formed by DNA damage [36,37]. In the λ*cI*^ind-^ lysogens, RecA mediated CI autocleavage is prevented, and the few phage produced are CI low expression mutants [36]. Indeed, free phage isolated from feces of mice colonized with λ*cI*^ind-^ lysogens formed clearer plaques than λ wild-type, which suggest they have a lower lysogenization rate. Sequencing of the *cI* gene from 9 phages isolated at day 2 either from free phage in feces or from S^L^ bacteria revealed they all had a point mutation in the -35 box of CI promoter, P_RM_ (G->T, -33 relative to the *cI* start of transcription). Interestingly, this P_RM_ mutation was previously shown to enable λ prophage induction in the absence of SOS activation, by decreasing by 80% intracellular CI levels, leading to much higher switching rates from the lysogenic to the lytic states [36]. Indeed, these P_RM_ mutants, named λcI*, had an induction rate 50,000-fold higher than that of the ancestral λ*cI*^ind-^ phage and 300-fold higher than the wild-type (Table 2). Measurement of induction rate from 12 other S^L^ bacteria revealed they were all lysogenized by λcI*. The high induction of this virulent mutant enabled its propagation during the first days of colonization. However, in agreement with evolutionary epidemiology theory, that predicts that selection for virulence decreases with the pool of susceptible hosts [38], the virulent λcI* mutant was counter selected later on in the prophage form, due to killing of its host through induction (Fig 6).

### High phage propagation permits *de novo* horizontal gene transfer detection

**Fig 7:**
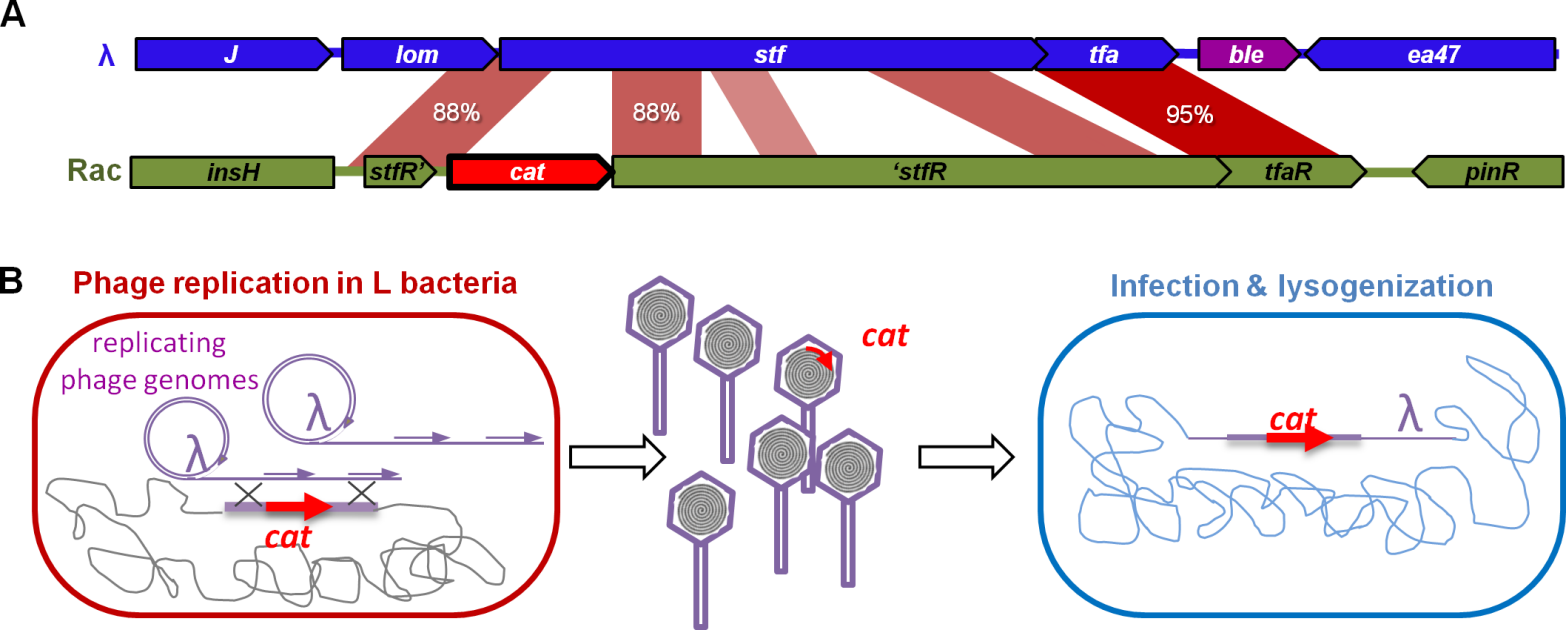
Horizontal gene transfer mediated by λ. **A)** The *cat* gene conferring chloramphenicol resistance Cm^R^ (red arrow, cat) of the lysogenic strain is inserted in the defective prophage Rac. The gene is flanked by regions that share homology with the phage lambda. **B)** Upon induction, the λ phage replicates and can recombine with the bacterial chromosome in such a way that the *cat* gene is captured by homologous recombination. Bacteria lysogenized by a recombinant phage are resistant to chloramphenicol.

We next investigated whether this high phage activity allowed for gene exchange between the phage and bacterial genomes. We have previously reported that λ captures bacterial genes by homologous recombination during the lytic cycle, at frequencies ranging between 10^−4^ and 10^−6^ depending on the extent of homology between the DNA segments (Fig 7 and [20]). In our experimental system, recombination can lead to the incorporation of the chloramphenicol resistance gene (*cat*) of the L strain into the phage genome, since L bacteria have the *cat* gene in a chromosomal region of partial homology with λ (88% identity). We investigated the occurrence of this phenomenon in mice. Recombinant phages can be detected in their lysogenic form as they confer chloramphenicol resistance to the bacteria they are integrated in. On days 1 and 2, recombinant prophages were detected in all mice, at frequencies around 5.10^−8^ relative to the number of new lysogens (S^L^). PCR analysis confirmed that the *cat* gene was placed at the expected position in the λ prophage. No recombinants were detected with a λ phage deleted of its main recombination gene, *bet* (or *redβ*), indicating the importance of phage recombination function for gene acquisition.

## Discussion

Complete genome sequencing of thousands of gut bacteria has shown that most harbor prophages, yet their impact on strain fitness in the gastrointestinal tract has rarely been investigated. Colonization experiments, supported by a mathematical model of phage/bacteria interactions, show that the advantage of λ lysogeny in monoxenic mice gut is valid only when susceptible bacteria are present; a situation that might be only occasional in the gut microbiota. Indeed, it is supposed that only one or two *E*. *coli* strains cohabit at the same time in the human gastrointestinal tract [39,40], and moreover, to our knowledge λ phage infects only a small proportion of *E*. *coli* strains. In the absence of susceptible competitors, the prophage was costly for its host, due to frequent induction caused by DNA damage. Prophages were generally shown to positively impact their host fitness, and our study is, to our knowledge, the first demonstration that a prophage can be detrimental to bacteria in the gastrointestinal tract.

The level of phage λ induction observed in monoxenic mice was remarkable: 1 to 2% of lysogenic bacteria were lysed *per* generation, which is almost two orders of magnitude higher than in standard laboratory conditions, in which induction was too low to constitute a measurable fitness cost (Figs 1C and S4). This result is reinforced by another study showing that the induction rate of 933W lambdoïd prophage is higher in the mouse gastrointestinal tract than *in vitro*, and constant over time [41]. However, the reporter assay used did not permit direct estimation of the induction rate and the associated cost for the bacteria [41]. We observed that the λ repressor mutation CI^ind-^, which abolishes CI auto-cleavage, dramatically decreased prophage induction in the intestine. Many reports over a long period of time have proven unambiguously that for such cleavage to occur, a RecA nucleoprotein filament (also called “activated RecA”) must catalyze the reaction [36,42,43], so lambda prophage induction reflects DNA damage. Since RecA nucleoprotein filament also triggers the general SOS response [44], our results indicates that this response is activated in 1 to 2% of bacteria in the intestine of monoxenic mice. DNA damage sensing is not only responsible for the induction of most prophages [2], but it also triggers activation of other mobile genetic elements such as integrative and conjugative elements or ICEs [45], transposons [46] and integrons [47]. Moreover, the large number of defective prophages in *E*. *coli* genomes, i.e. prophages incapable of independent induction or particle formation [48–51], suggests a regular selection in favor of bacteria having lost or inactivated these prophages, possibly in response to their frequent induction in the intestine. Interestingly, at least in the simplified gut environment used in this study, induction cost is not compensated by the putative adaptative genes *lom*, *bor* and *rex* described in the λ genome [52,53]. The low induction rate λ*cI*^*ind-*^ phage used in this study was on the contrary beneficial to its host, since it confers no induction cost while still enabling efficient killing of competitors through amplification on susceptibles. However, phage mutants with higher induction are strongly selected for when susceptible bacteria are present [38], as exemplified in our study by the selection of the λcI* virulent phage (Fig 6). Interestingly, all *E*. *coli* lambdoïd phages tested have comparable levels of induction [54], close to that of lambda, suggesting they have all evolved toward the same optimal induction rate for propagation. Alternatively, it was proposed that optimal induction rates evolved to benefit the bacterial host–and thereby prophage vertical transmission- by a “bacterial altruism” mechanism [55]. Indeed, in the case of Shiga-toxin carrying prophages, prophage induction leads to the release of toxins killing bacterial protozoan predators, benefiting the bacterial host even in the absence of susceptible competitors.

The level of phage λ lysogenization unraveled was also remarkable: we estimated that in monoxenic mice gastrointestinal tract the lysogenization rate is close to 20%. By comparison, *in vitro*, lysogenization rate is close to 0.1% in bacteria growing rapidly [54], but almost 50% in starved bacteria [25,56]. This lysogenization rate estimated in mouse is therefore much higher than generally measured or assumed [16,17,57,58]. Since the final gain of the original phage carrier on the susceptible strain is inversely proportional to the lysogenization rate (our results and [57]), in the gastrointestinal tract high lysogenization results in a smaller gain of the original phage carrier than previously described. Theory predicts that high lysogenization optimizes phage reproduction in an environment where the density of susceptible hosts is low or variable [58,59]. Although the gut microbiota is the densest bacterial community on earth, it includes hundreds of different species and thousands of bacterial strains, possibly making highly specific phage infection relatively rare. Low phage susceptibility seems to be the conclusion of a large-scale study of phage-bacteria interactions in a gnotobiotic mouse model [60]: in mice raised with a simplified microbiota composed of 15 strains belonging to dominant human species, only two were attacked by a cocktail of thousands of different phages isolated from a human gut microbiota. Moreover, a higher proportion of temperate phages was found in gut viromes than in other environments [19,61]. Altogether, these data support our results, suggesting that in the gastrointestinal tract the lysogenic life cycle of phages is favoured compared to lytic multiplication.

Temperate phages being major actors of horizontal gene transfer in bacteria, a concern emerged recently regarding their role in the propagation of antibiotic resistance genes [62]. Indeed, some phage particles are vectors of antibiotic resistance genes [28]. Most of the time, gene transfer occurs by generalized transduction, the erroneous encapsidation of bacterial DNA. Such errors are rare: for instance, the proportion of *E*. *coli* phage P1 capsids leading to the production of an antibiotic-resistant clone is between 10^−5^ and 10^−6^ [63,64]. The incorporation of a bacterial gene into phage genome, and afterwards transfers by lysogenization is a much rarer event, that we could detect only when the gene was located in a defective prophage sharing homology with λ [20]. In the present study, we estimated the frequency of such gene capture (*cat* gene, conferring chloramphenicol resistance) by λ in mice to be 10^−8^. Interestingly, up to now very few cases of phages encoding resistance genes have been reported [65–67], suggesting that even if the lysogenization rate in the intestine is very high, the risk of antibiotic resistance spread mediated by temperate phage is low.

Here we found that in monoxenic mouse gastrointestinal tract, lysogeny initially benefits its host during competitions with susceptible bacteria, in line with previous studies in other environments [16–18]. The mathematical model highlighted that the benefit of the original lysogenic strain depends critically on the lysogenization and induction rates: the lower these parameters, the higher the benefit. We also show that in monoxenic mice gastrointestinal tract, DNA damage leads to high prophage induction, which results in a significant cost for the lysogen. Provided that DNA damage observed in monoxenic mice gut also occurs in conventional animals, since all *E*. *coli* lambdoïd phages tested have comparable levels of induction [54] -and since most *E*. *coli* prophages are lambdoïd [68]- our results might prove to be general. Due to the highly specific phage-bacteria interactions, we hypothesize that the absence of bacteria susceptible to a particular phage in the gastrointestinal tract might regularly occur, and that on the long term, the parasitic aspect of at least some active prophages prevails.

## Materials and Methods

### Bacterial strains

All bacterial strains are described in S1 Table. All strains were constructed by modifying the MG1655 Δ*fliC* Δ*ompF* strain. This strain was used because *ompB* mutations are rapidly and systematically selected in the MG1655 strain in the mouse gut as a result of their effects on flagellin (FliC) repression and of decreased membrane permeability *via* repression of the major porin OmpF [69]. As *ompB* mutants also display a reduced expression level of LamB [31], a maltoporin used by phage λ for infection, we used a *ΔompF ΔfliC* strain in which no *ompB* mutations were selected [69]. The *stfR*::*cat* mutation was introduced in this strain by phage P1 transduction from the MD19 strain described in [20]. The Δ*lamB* strains were constructed by phage P1 transduction of the *lamB*::*KanR* cassette from the Keio collection strain JW3996 [70]. In the MD56 and MD74 strains, the KanR cassette was excised as described in [71]. The λ receptor being absent in Δ*lamB* strains, λ prophage was introduced by transformation with Urλ*ble* purified DNA.

### Phage strains

The λ*ble* phage strain used in this study was constructed by insertion of the phleomycin resistance gene *ble* into the Urλ strain of λ, as described in [20]. The λ*cI*^ind-^ mutant contains a mutation (A111T) in the RecA cleavage site: the alanine in position 111 is replaced by a threonine [72]. This mutation was introduced by recombineering with the oligonucleotide AT111 (GTAAAGGTTCTAAGCTCAGGTGAGAACATgCCgGttTGgACATGAGAAAAAACAGGGTACTCATACC). Small letters represent changes in the DNA sequence. Several neutral differences were added to the one necessary for the amino acid change in order to avoid recognition by MutS. Recombineering was performed in the HME57 strain [73], which carried plasmid pKD46 [71], and lysogenized with λ*ble*. The strain was co-transformed with two oligonucleotides, AT111 and Court’s lab oligonucleotide 144, conferring it the ability to use galactose [74]. After transformation, colonies were isolated on M9 minimal galactose plates. 96 Gal+ clones were screened for the absence of spontaneous phage induction, by scoring the absence of infectious phage particles in culture supernatants. 1 out of 96 clones had the expected mutation, which was confirmed by sequencing of the *cI* gene. The λ*cI*^*ind-*^ phage was next introduced into the MG1655 Δ*fliC* Δ*ompF stfR*::cat strain by P1 transduction and selection on phleomycin plates.

### Mouse and in vivo competition experiments

Germ-free C3H/HeN mice were bred at the germ-free animal facilities of the INRA Micalis Institute, Anaxem. Mice were reared in isolators and fed *ad libitum* on a commercial diet sterilized by gamma irradiation (40 kGy) and supplied with autoclaved tap water. For colonization experiments, 8 week-old germ-free female mice were gavaged with 10^6^ bacteria from the chosen strain, or the appropriate mixture of the two strains, in 0.1 mL of M9 minimal medium. The cassettes used to differentiate strains during competition confer resistance to chloramphenicol or to kanamycin. Their expression is known to have no significant cost during *E*. *coli* intestinal colonization, so no inversion of markers was performed [31,75]. Bacterial and phage populations in feces were monitored by colony forming unit (CFU) and plaque forming unit (PFU) counts in freshly harvested individual fecal samples, as described below. Feces were homogenized in a 10-fold volume of sterile water before dilution in LB and plating on LBA plates with the appropriate antibiotics. PFUs were enumerated in the supernatant of suspended feces centrifuged 3 minutes at 12,000 g. All procedures were carried out in accordance with the European guidelines for the care and use of laboratory animals. The project received the agreement of the local DDPP (n° A48-195) and from the local ethic committee for animal experimentation, the Comethea (n° 13–05).

### Phage and bacterial counts

After serial dilutions, bacterial populations were determined by plating on selective antibiotic-LB agar plates (1.5% agar). Antibiotics were used at the following concentrations: kanamycin (50 μg/mL), chloramphenicol (20 μg/mL), and phleomycin (5 μg/ml). PFUs were determined by spotting 10 μl of serial dilutions of diluted feces on a lawn of the indicator bacteria in top agar (0.4% agar, 10 mM MgSO_4_). The indicator bacterial culture was fresh MD5 culture grown in LB containing 0.2% maltose. Latent bacteria were counted similarly but after elimination of free phage by centrifugation. CFUs and PFUs were counted after 12–16 hours of incubation at 37°C. The ability to use maltose was monitored in tetrazolium maltose (TM) indicator plates. Mal^+^ and Mal^-^ clones respectively form white and red colonies on these plates. The TM medium was composed of tryptone (10 g/L), yeast extract (1 g/L), NaCl (5g/L), agar (16g/L), maltose (5 g/L) and tetrazolium dye (50 mg/L, Sigma).

#### S and L lineages competition *in vitro*

For comparison with the *in vivo* results, S and L strains were co-cultured *in vitro* in rich LB medium, with a starting 1:1 ratio. Co-cultures were diluted 1,000-fold twice a day in 10 ml of LB, with or without 5 mM MgSO_**4**_, in 50-ml plastic tubes and incubated at 37°C with agitation (200 rpm). 1,000 fold dilution corresponds to a number of bacterial generations per 24 hours comparable to that in mouse gut (about 20 generations compared to 16 in mice).

### Adsorption rate measurements

The technique was essentially that of Hendrix [76], with minor modifications. Adsorbing bacteria from feces were prepared as described for enumeration. A control culture was grown at 37°C with shaking in LB + 0.2% maltose + 10 mM MgSO_4_ up to an absorbance at 600 nm of 1.2 (about 6.10^8^ bacteria/ml). Aliquots of 200 μL of culture were added to 50-μL aliquots containing 500 phage particles in 400-μL PCR tube strips, and mixed at 37°C. Several strips were prepared, one for each time point. At the chosen time points, the bacteria were separated from the free phages by centrifugation. The PFU in the supernatants were enumerated as described above. PFUs at time zero were estimated by titering the phage suspension. The slope of a graph plotting the logarithm of the number of bacteria remaining unadsorbed as a function of time allowed us to calculate the adsorption rate a according to the equation N_t_ = N_0_ x e^-Bat^, where N_t_ and N_0_ are the numbers of phage particles unadsorbed at time t and at time zero respectively, B is the number of bacteria per millilitre, and t is time in hours.

### Quantitative estimation of mathematical model’s parameters

To calibrate the mathematical model, we used different datasets and versions of the model, aiming at the disentanglement of the eight parameters (see S1 Text for details). We identified four groups of parameters, which were estimated sequentially. The first group consists of parameters *r*, *k* and *d* that directly control the general growth of bacterial populations. As a global parameter, independent of the bacterial strains, the dilution rate was fixed to *d* = 0.25, in line with [77–79]. Subsequently, parameters *r* and *k* were estimated from single species experiments in mice, using a mono-dimensional logistic equation and nonlinear least square minimization. The second group consists of parameters *x* and *l*. A closer look at the equations points out the central role of these two parameters on the dynamics of latent cells Q. To further separate the estimation from phage-dependent parameters, we considered a LamB^-^ version of the model (i.e. with a = 0) implying only variables L, S and Q. This simplified model was then fitted to LamB^-^ experimental data. The third group gathers phage-dependent parameters *y* and *a*. Based on the formula for the equilibrium population of free viruses, a linear relationship was obtained between the two. Using a value of *y* calculated from *in vitro* data, the corresponding value for the adsorption constant was deduced. Finally, the last parameter *g* (probability of lysogenization) was estimated by fitting the ratio L/(S+S^L^) in L-S competition experiments. The best fit was obtained at 36 h. All estimated values are listed in Table 1. When possible, bootstrap techniques were used to compute 95% confidence intervals (see S1 Text). All numerical computations have been performed with Matlab (The MathWorks, Inc.).

### Experimental estimation of burst size

Burst size was estimated *in vitro* in conditioned LB, i.e. the supernatant of a bacterial culture grown in LB (+0.2% maltose, +10 mM MgSO_4_) at 37°C up to an absorbance at 600 nm of 1.5. In such conditioned media we measured a growth rate of 1.1 h^-1^, close to that measured in the mice gastrointestinal tract during the first 24 hours of colonization. For single burst experiments, 100 μl of a culture at OD600 of 1.5 is mixed with 1x10^5^ λ phage. The mix of phage and bacteria is incubated 7 minutes at 37°C, centrifuged and washed twice to eliminate free phage, and then diluted 1,000 times in conditioned LB at 37°C. Samples were taken every 10 minutes, mixed with 200 μl of exponentially growing susceptible bacteria and 3ml of Top Agar (4.5 g/L, 0.2% maltose, 10 mM MgSO_4_), and then poured on LB agar plates. Plaque-forming units (PFU) were counted. The burst size (y) is the factor between maximum and initial PFU counts. We determined that y = 12.1 ± 8.

### Model-independent estimation of induction rate

Since induction rate is the sole parameter differentiating growth of S and L *lamB* lineages, the induction rate (x) was determined from the evolution of the L/S ratio between days 0 and 9 in function of the number of generations. The number of generations was estimated by assuming that growth rate is equal to r, 1.1 h^-1^, during the first 24 hours, and after equal to excretion rate d, 0.25 h^-1^. A linear regression on data from 6 mice (lm function in R software) gave x = 0.0170 ± 0.0046.

### In vitro induction rate measurements

We adapted a method allowing for an measurement of the induction rate *per* bacterium [80]. Lysogenic bacteria were diluted 200 fold in LB and grown at 37°C with shaking. When specified, 0.8% w/v bile salts (cholic acid-deoxycholic acid sodium salt mixture, sigma-aldrich) were added when the absorbance at 600 nm of the culture was 0.2 or 0.1. When the absorbance reached 0.4, cultures were swirled on ice for 5 minutes and washed twice at 4°C to eliminate free phages. Washed lysogenic bacteria were then mixed at the appropriate dilution with 100 μl of a saturated culture of the indicator strain, 3 ml of top agar were added, and the mix was plated on LBA-ampicillin plates (50 μg/ml). The indicator bacteria were ampicillin-resistant (strain JC10990 *recF*::Tn3 AmpR). Ampicillin prevents further lysogen growth but does not prevent the completion of the lytic cycle if already started, which results in an infective centre. The induction rate was calculated by dividing the number of infective centres by the number of plated lysogenic bacteria.

## Supporting Information

S1 Fig Map of the six mutations identified in the *malT* gene. 3 mutations interrupt the coding sequence, resulting in a truncated protein. The three other mutations are found in the maltotriose sensor domain DT3, and two have been selected independently, as they are found in two mice from two different cages. Interestingly, 24 out of 29 point mutations in this domain were observed to abolish of very significantly diminish MalT activity [81].
(PDF)

S2 Fig Evolution of the ratio of lysogen over susceptible lineages (L/(S+S^L^)) over time according to the model, for two values of the burst size. **A**) burst size of 12.1. **B**) burst size of 3. In this last situation, the slope is not as steep as in panel A.
(PDF)

S3 Fig Sensitivity analysis. Evolution of the final ratio of lysogen over susceptible lineages (L/(S+S^L^)), approximated using numerical simulations of the mathematical for t = 100 h, when each parameter is perturbed individually within the range indicated on the x axis. The three colors correspond to three different initial ratios (see upper left panel).
(PDF)

S4 Fig L over S lineages ratio. **A)** Evolution of the ratio of lysogen over susceptible lineages (L/(S+S^L^)) with time in broth (LB). A clear advantage of the lysogenic strain in observed in LB + Mg^2+^. With *lamB* strains, the ratio is completely stable, showing that no cost associated to prophage induction is detectable. Very similar results are observed in LB without Mg^2+^. Mean +/- standard deviation of ratios on 4 independent cultures. **B)** Same ratio in mice with *lamB* bacterial strains and λ*cI*^ind-^ phage. Mean +/- standard deviation of ratios on 3 mice.
(PDF)

S1 Text Mathematical modelling of phage-mediated bacterial interactions in the mouse gut. Part 1 contains a description of the model's construction together with a brief analysis of its equilibrium points. Part 2 details the procedures used to estimate the model's parameters. Part 3 proposes a sensitivity analysis of the parameters.
(PDF)

S1 Table Bacterial and phage strains used in the study. (DOCX)
