# Supplementary figures and images for: Carriage of λ Latent Virus Is Costly for Its Bacterial Host due to Frequent Reactivation in Monoxenic Mouse Intestine
Source: PLoS Genet. 2016 Feb 12;12(2):e1005861. doi: 10.1371/journal.pgen.1005861 (PMC4752277; doi:10.1371/journal.pgen.1005861)

Figure S1

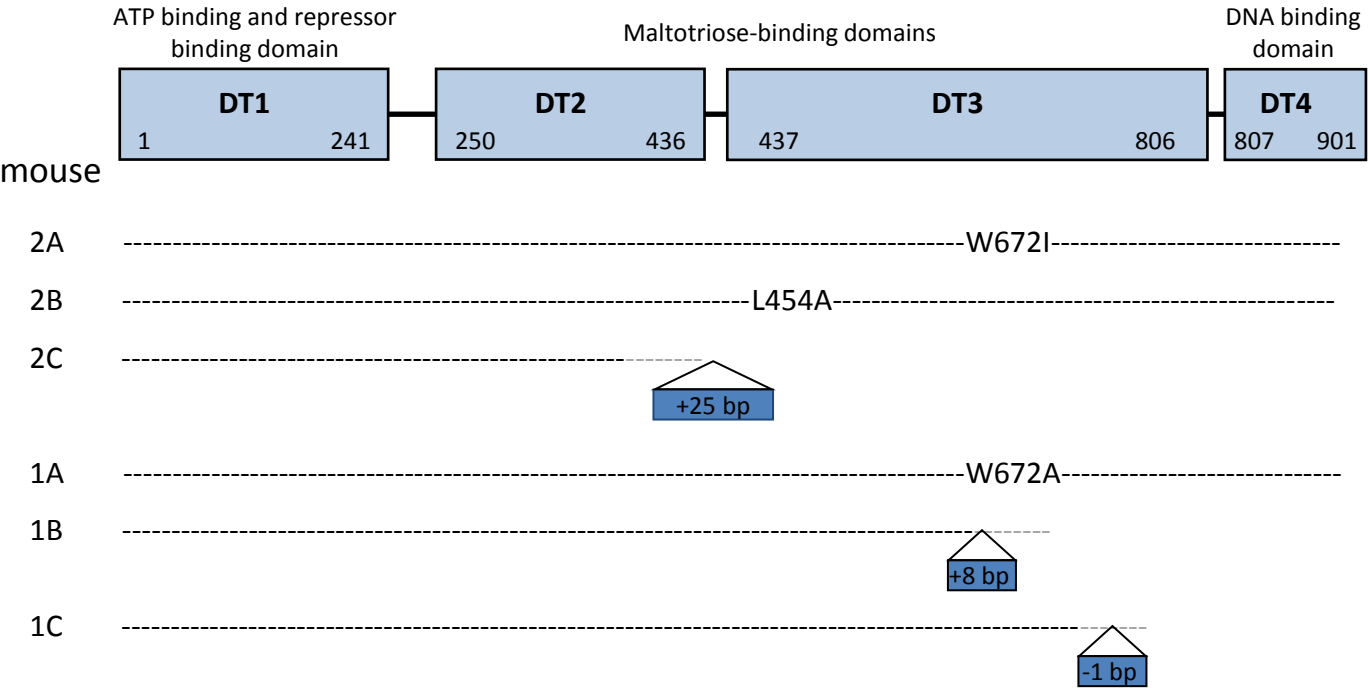

Supplement: S1 Fig — 3 mutations interrupt the coding sequence, resulting in a truncated protein. The three other mutations are found in the maltotriose sensor domain DT3, and two have been selected independently, as they are found in two mice from two different cages. Interestingly, 24 out of 29 point mutations in this domain were observed to abolish of very significantly diminish MalT activity [81]. (PDF) [file pgen.1005861.s001.pdf]

Figure S2

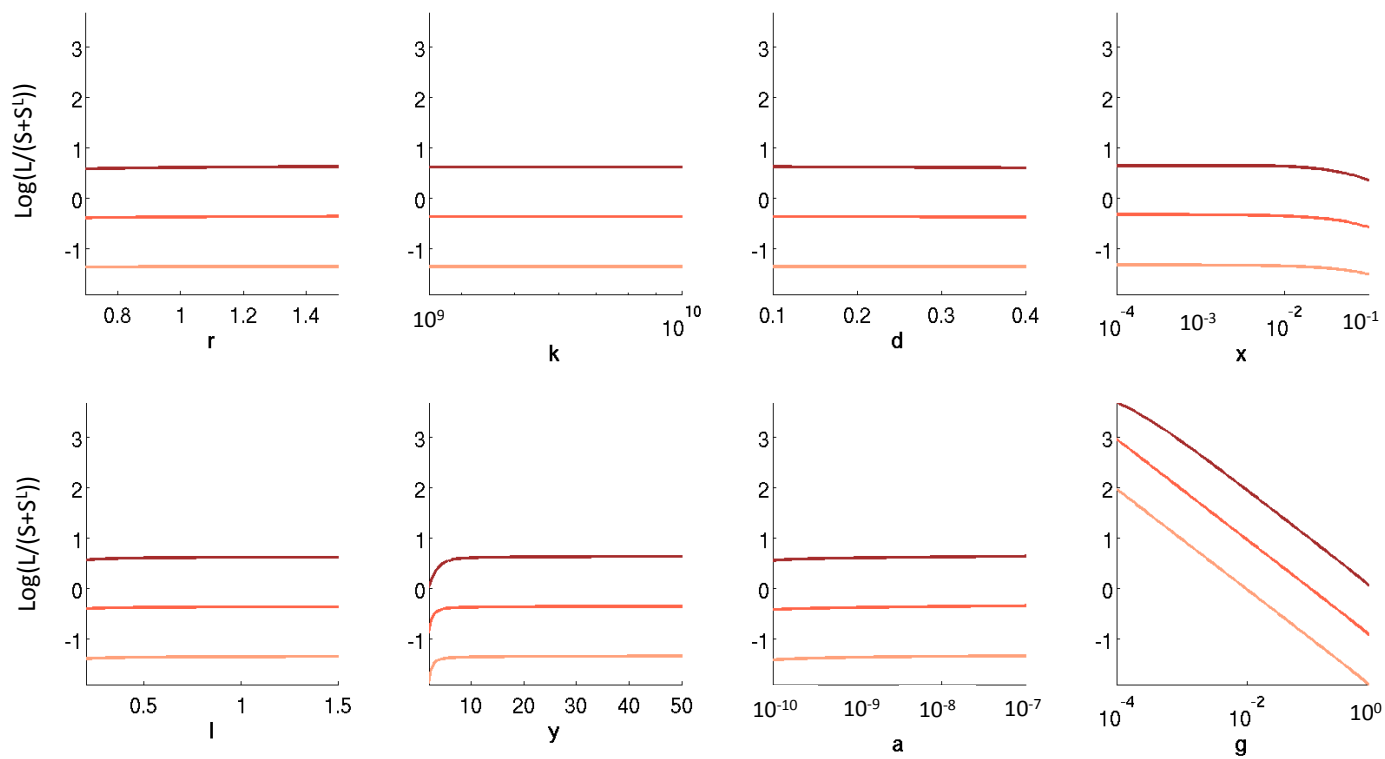

Supplement: S2 Fig — A) burst size of 12.1. B) burst size of 3. In this last situation, the slope is not as steep as in panel A. (PDF) [file pgen.1005861.s002.pdf]

Figure S3

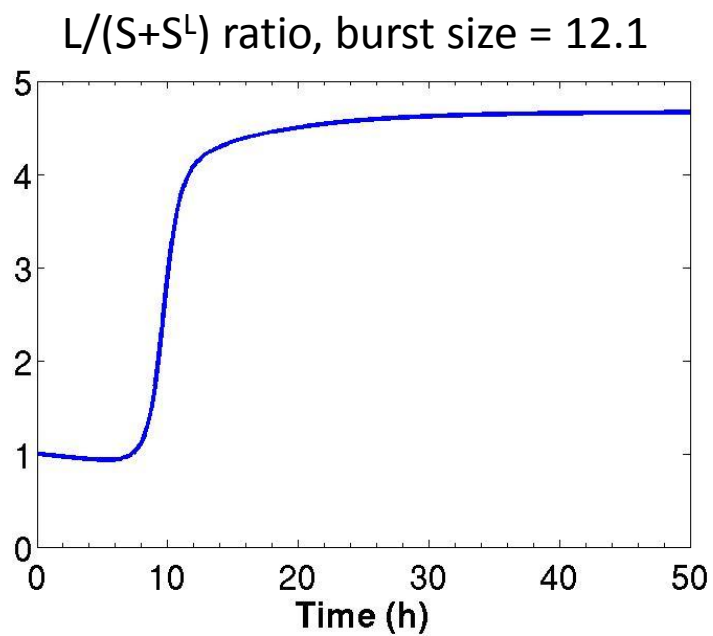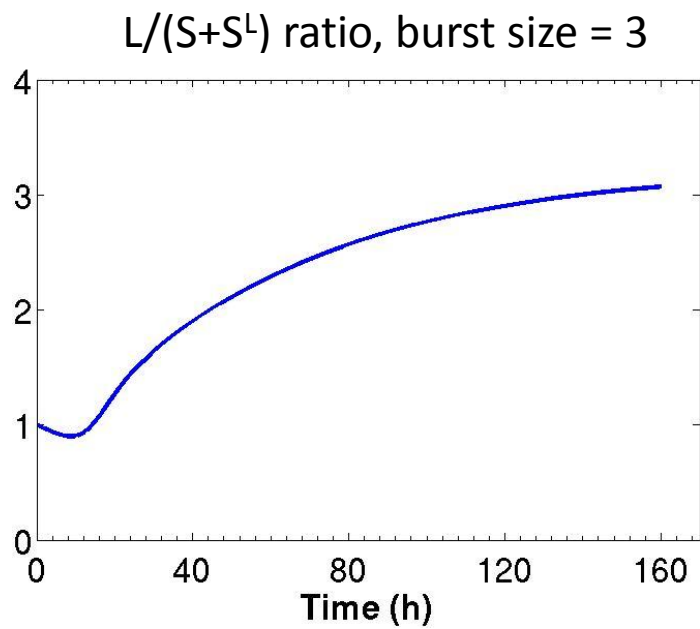

Supplement: S3 Fig — Evolution of the final ratio of lysogen over susceptible lineages (L/(S+SL)), approximated using numerical simulations of the mathematical for t = 100 h, when each parameter is perturbed individually within the range indicated on the x axis. The three colors correspond to three different initial ratios (see upper left panel). (PDF) [file pgen.1005861.s003.pdf]

Figure S4

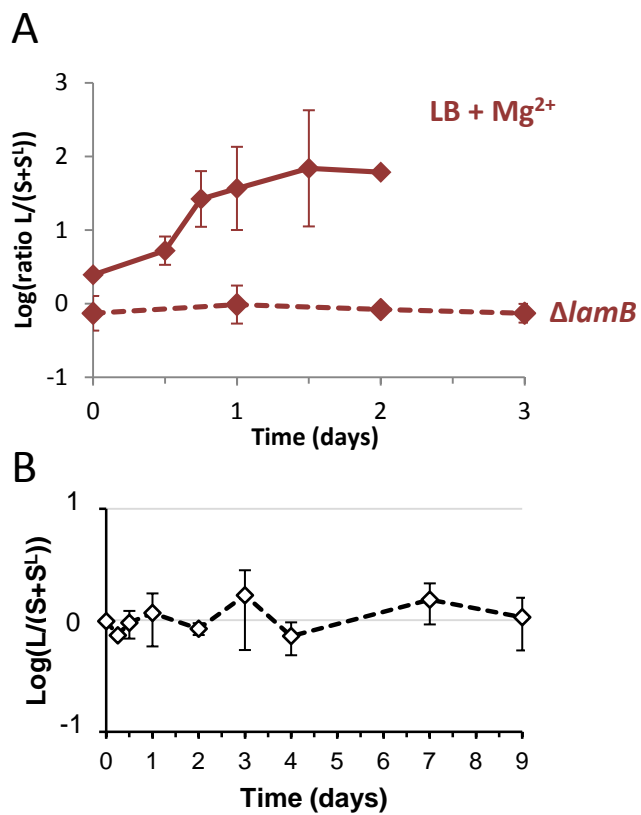

Supplement: S4 Fig — A) Evolution of the ratio of lysogen over susceptible lineages (L/(S+SL)) with time in broth (LB). A clear advantage of the lysogenic strain in observed in LB + Mg2+. With lamB strains, the ratio is completely stable, showing that no cost associated to prophage induction is detectable. Very similar results are observed in LB without Mg2+. Mean +/- standard deviation of ratios on 4 independent cultures. B) Same ratio in mice with lamB bacterial strains and λcIind- phage. Mean +/- standard deviation of ratios on 3 mice. (PDF) [file pgen.1005861.s004.pdf]
