# Supplementary material for: Carriage of λ Latent Virus Is Costly for Its Bacterial Host due to Frequent Reactivation in Monoxenic Mouse Intestine
Source: PLoS Genet. 2016 Feb 12;12(2):e1005861. doi: 10.1371/journal.pgen.1005861 (PMC4752277; doi:10.1371/journal.pgen.1005861)
Supplement: S1 Text — Part 1 contains a description of the model's construction together with a brief analysis of its equilibrium points. Part 2 details the procedures used to estimate the model's parameters. Part 3 proposes a sensitivity analysis of the parameters. (PDF) [file pgen.1005861.s005.pdf]

S1 TEXT. Mathematical modelling of phage-bacteria interactions in the mouse gut

1 Model construction and analysis

1.1 Phage-mediated bacterial competition in monoxenic mouse gut

To model the dynamics of different microbial populations in the mouse gut, we built a 5-dimensional ordinary differential equation (ODE) system, inspired from [Brown *et al*, 2006]. The temporal evolution of three bacterial populations is considered: population L (lysogens) is carrying the prophage, population S (susceptible) is devoid of it, and population S^l (lysogenized susceptible) is the newly-infected susceptible population (it is assumed initially $S^l(0) = 0$). Total population is denoted by $N = L + S + S^l$. Both lysogenic populations (L and S^l) are able to enter phage-mediated lysis, which in the model is represented by a transient phase of latency (population Q). At the end of this phase, the cell dies and free viruses (V) are released in the medium. The four bacterial populations L, S, S^l and Q as well as the viral population V are expressed as cell densities (respectively, in CFU/g and in PFU/g). These five variables evolve according to the following system of ODE:

$$(\mathcal{S}) \qquad \begin{aligned} \frac{dL}{dt} &= r\left(1-\frac{N}{k}\right)L - xL - dL, \\ \frac{dS}{dt} &= r\left(1-\frac{N}{k}\right)S - aVS - dS, \\ \frac{dS^l}{dt} &= r\left(1-\frac{N}{k}\right)S^l - xS^l + gaVS - dS^l, \\ \frac{dQ}{dt} &= x\left(L+S^l\right) + (1-g)aVS - lQ - dQ, \\ \frac{dV}{dt} &= ylQ - aV(N+Q) - dV. \end{aligned}$$

System $(\mathcal{S})$ has eight parameters, listed in Table A1 below, all supposed positive. It is built upon classical modelling assumptions (see [Brown *et al*, 2006]), and it is adapted to take into account the mouse gut environment used in our study.

First, populations L, S and S^l are assumed to grow according to logistic terms, with a common maximal growth rate r (h^{-1}) and a common carrying capacity k (CFU/g). While empirical, the logistic equation is an efficient mathematical model to represent microbial dynamics. Using common growth parameters r and k for both populations is a good way to assess their competition over a common resource. From a biological point of view, this assumption seems reasonable as both L and S strains are identical except for the presence of the prophage, and it is further confirmed by *in vitro* experiments in LB (see Fig. 1D). Using a similar argument, the fraction of lysogenic bacteria (L and S^l) entering phage-induced lysis are supposed to happen at a common rate x (h^{-1}). Classically, phage-mediated infection of susceptible bacteria occurs at a rate determined by a constant a ($[PFU/g]^{-1}h^{-1}$) called the adsorption constant [Delbruck, 1940], which depends on the phage and its host. In the model, the binding of a bacterium and a phage eventually leads to only two possible outcomes: lysogenization with probability g and direct lysis with probability $1-g$. Finally, parameter l determines the latency rate, *i.e.* the mortality rate of latent cells, and parameter y is the average number of free viruses released at the end of the lytic cycle (burst size). With respect to the model in [Brown *et al*, 2006], several adjustments have been made, to take into account new constraints and new hypotheses. First, each equation has an additional linear dilution

term with a common rate d (h^{-1}) to account for the fact that the populations evolve within monoxenic mouse gut. Second, the disappearance of free viruses, represented by the term $-V\big(d+a(N+Q)\big)$, is based on two hypotheses: (*i*) free viruses are supposed to bind to viable cells N as well as to latent cells Q (in the latter case, having no other effect than the disappearance of the virus itself) and (*ii*) free virus mortality is ignored (it is assumed to be negligible, notably with respect to the dilution term). As always in such approaches, modelling choices mainly consist in compromises between the complexity of the biological phenomenon, the level of detail available from experimental data and the practicability of the mathematical analysis. In the present study, a particular effort was devoted to the integration of quantitative temporal data, in order to provide a quantitative estimation of most of the eight parameters (see Part 2 below), as close as possible to our experimental setup.

Table A1. List of parameters of the mathematical model.

	Description	Unit	Comments
d	Dilution rate of mouse gut	h^{-1}	Global parameter, assumed independent of bacterial competition.
r	Maximal growth rate	h^{-1}	Bacterial growth parameters, assumed independent of phage and phage-mediated infection.
k	Carrying capacity	CFU/g	
x	Induction rate	h^{-1}	Parameters governing phage-mediated bacterial lysis.
l	Latent cells mortality rate	h^{-1}	
y	Burst size	∅	Parameters governing bacterial infection by the phage.
a	Adsorption constant	$[\mathrm{PFU/g}]^{-1}h^{-1}$	
g	Probability of lysogenization	∅	

1.2 Analysis of equilibrium points

To give a brief analysis of dynamical system ($\mathcal{S}$), we propose a symbolic computation of its equilibrium points, in function of the eight parameters. For each one, symbolic conditions over the parameters are derived for the equilibrium to be admissible (since variables are densities, only nonnegative values are acceptable). All parameters are supposed positive and g, being a probability, belongs to $[0,1]$. The stability was not analyzed symbolically in each case, but for each equilibrium we further exhibit a set of parameter values for which the equilibrium is numerically stable. Simulations were performed with Matlab (The MathWorks, Inc.), using `ode45` solver. For the sake of conciseness, only the main results of the symbolic computations are reproduced here.

First, observe that since the same logistic growth term is used for populations L, S and S^l, a new differential equation can be derived for variable N. By substituting it to the equation for L, we obtain an equivalent formulation of the system:

$$\begin{aligned}
dN/dt &= r(1-N/k)N - x(N-S) - (1-g)aVS - dN,\\
dS/dt &= r(1-N/k)S - aVS - dS,\\
dS^l/dt &= r(1-N/k)S^l - xS^l + gaVS - dS^l,\\
dQ/dt &= x(N-S) + (1-g)aVS - lQ - dQ,\\
dV/dt &= ylQ - aV(N+Q) - dV.
\end{aligned}$$

In this formulation, variable S^l does not affect the other variables of the system, thus reducing the analysis to the search of the equilibria of the following 4-dimensional system:

$$\frac{dN}{dt} = r\left(1-\frac{N}{k}\right)N - x(N-S) - (1-g)aVS - dN,$$
$$\frac{dS}{dt} = r\left(1-\frac{N}{k}\right)S - aVS - dS,$$
$$\frac{dQ}{dt} = x(N-S) + (1-g)aVS - lQ - dQ,$$
$$\frac{dV}{dt} = ylQ - aV(N+Q) - dV.$$

Let X designate a vector (N, S, Q, V) and F designate the vector field of the previous system. The search for equilibria is made in two main steps. As a first step, the symbolic resolution of the equation $F(X) = 0$ leads to the existence of potential equilibria. In a second step, we investigate whether those solutions lead to an admissible equilibrium, where each population is nonnegative. In other words, we check the following set of constraints:

$$(\mathcal{A}):\ \{0 \le S \le N, 0 \le Q, 0 \le V\}.$$

First, observe that the equation $\frac{dS}{dt} = 0$ leads to two cases: either *(i)* $S = 0$, or *(ii)* $r(1 - N/k) = aV + d$.

Case (i) leads to two possible equilibria. The first one, denoted by X^0 is given by:

$$L = S = S = Q = V = 0.$$

This is always an admissible solution and it corresponds to a "wash-out" situation, where all species eventually disappear. A symbolic analysis of its stability can be made: it is asymptotically stable whenever $r < d$ (*i.e.* when bacterial growth is insufficient with regards to the dilution). The second possible equilibrium is denoted by X^L: it corresponds to a situation where susceptible bacteria eventually disappear in favor of lysogens L and S^l. It is admissible whenever $r > d + x$, and is given by:

$$N = L + S^l = k\left(1 - \frac{x+d}{r}\right), S = 0, Q = \frac{xN}{l+d} = \frac{xk}{l+d}\left(1 - \frac{x+d}{r}\right),$$
$$V = \frac{\frac{ylx}{l+d}N}{a\left(1+\frac{x}{l+d}\right)N + d} = \frac{ylxk}{a(l+d+x)k\left(1-\frac{x+d}{r}\right) + d(l+d)}\left(1 - \frac{x+d}{r}\right).$$

Now let's analyze case *(ii)*. Symbolic computations lead to the resolution of the following equation in N:

$$\left(N - k\left(1 - \frac{d}{r}\right)\right) \times (p_2N^2 + p_1N + p_0) = 0.$$

Coefficients p_i are: $p_2 = ar$, $p_1 = ak[l(y-1) - r]$ and $p_0 = -dk(l + d)$. Again, the resolution of this equation leads to two possibilities. The first one eventually gives the following equilibrium, denoted by X_1^S:

$$S = k\left(1 - \frac{d}{r}\right), L = S^l = Q = V = 0.$$

This equilibrium corresponds to a situation where susceptible bacteria completely take over, with the eventual disappearance of lysogens L and S^l. It is admissible whenever $r > d$.

Finally, the last case to consider is the resolution of $p_2N^2 + p_1N + p_0 = 0$. Given the signs of coefficients p_i, this polynomial always has a positive root, denoted by N^*. It further leads to a potential equilibrium, denoted by X_2^S and given by:

$$S = \frac{1}{1-g}\frac{B-N^*}{A-N^*}N^*, Q = \frac{r}{k(l+d)}(C-N^*), V = \frac{r}{ak}(C-N^*),$$

where constants A, B, C are defined by:

$$\begin{cases} A = k\left(1 - \frac{d + x/(1-g)}{r}\right), \\ B = k\left(1 - \frac{d+x}{r}\right), \\ C = k\left(1 - \frac{d}{r}\right). \end{cases}$$

In X_2^S, population S survives, but does not take over. Further numerical explorations seem to indicate that L eventually disappears (Figure A1-D), meaning that the S strain survives under both forms: uninfected S and lysogenized S^l. To analyze its admissibility, we have to distinguish two situations:

- Suppose $r > d + x$. Then, X_2^S is admissible if and only if two conditions are satisfied:
$$\begin{cases} ak(r-d-x)[l(y-1)-d-x] < rd(l+d), \\ ak(r-d)[l(y-1)-d] > rd(l+d). \end{cases}$$
- Suppose $d < r < d + x$. Then, X_2^S is admissible if and only if:
$$ak(r-d)[l(y-1)-d] > rd(l+d).$$

Previous inequalities can be equivalently expressed as conditions over the burst size y:

$$ak(r-d)[l(y-1)-d] > rd(l+d) \Leftrightarrow y > y_1 := 1 + \frac{1}{l}\left(\frac{rd(l+d)}{ak(r-d)} + d\right),$$
$$ak(r-d-x)[l(y-1)-d-x] < rd(l+d) \Leftrightarrow y < y_2 := 1 + \frac{1}{l}\left(\frac{rd(l+d)}{ak(r-d-x)} + d + x\right).$$

To sum up, from the perspective of lineages L and S, four steady states may exist, illustrated numerically in Figure A1. In X^0, both strains eventually disappear (panel A). In X^L, the strain L takes over and susceptible only survive as new lysogens S^l (panel B). In X_1^S and X_2^S, the strain S takes over, either with complete phage disappearance (X_1^S, panel C), or with the coexistence of lysogenized S^l and uninfected S (X_2^S, panel D).

As evoked previously, the formal stability analysis of those equilibria has not been performed to its completion. However, early computations together with numerical experiments seem to indicate that there exists only one stable steady state for each set of parameter values. Stability conditions can be summarized according to the relative positions of r, d and x on the one hand, and to the relative positions of y, y_1 and y_2 on the other:

$r < d$	X^0 is the only stable equilibrium (wash-out).		
$d < r < d + x$	If $y < y_1$, X_1^S is the only stable equilibrium.	If $y > y_1$, X_2^S is the only stable equilibrium.	
$d + x < r$	If $y < y_1$, X_1^S is the only stable equilibrium.	If $y_1 < y < y_2$, X_2^S is the only stable equilibrium.	If $y > y_2$, X^L is the only stable equilibrium.

Despite this variety of asymptotic behaviors, typical parameter values for monoxenic mouse gut seem to always fall into the region where X^L is the only stable steady state ($r \gg d$ and $y \gg y_2$). Numerical parameter estimation from temporal data in mice confirmed this observation, as shown in Part 2 below. Therefore from now on, the term steady state will refer to equilibrium point X^L given above.

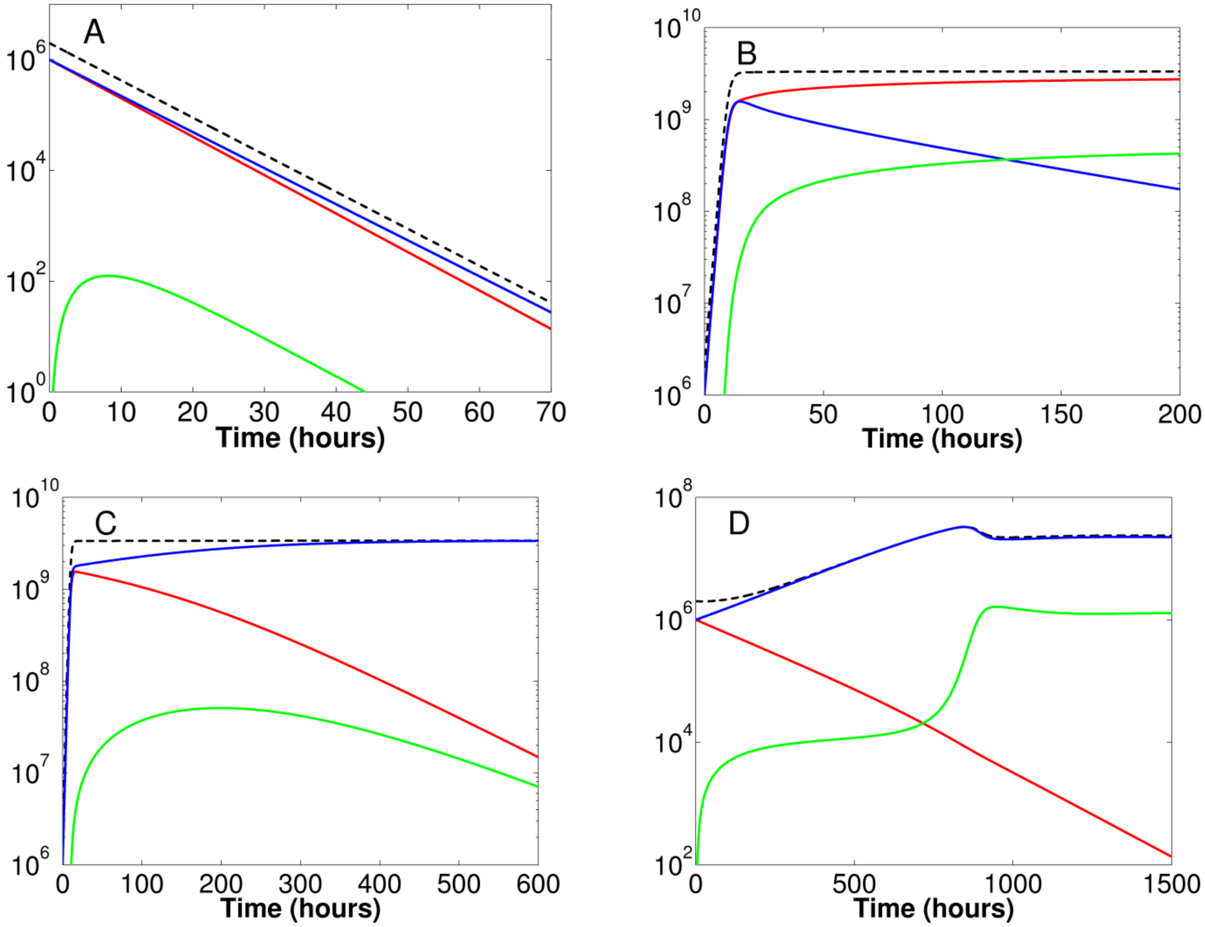

Figure A1. Numerical simulations of $(\mathcal{S})$, exhibiting the four possible equilibria. In each case, the initial condition is the same ($L_0 = S_0 = 10^6$) and common parameters value are: $d = 0.25$, $k = 4.5e9$, $a = 5e-9$, $g = 0.1$, $x = 0.01$ and $l = 1$. Red, blue and green correspond respectively to L, S and S^l, and the black curve corresponds to total population N. Panel A: wash-out X^0 ($r = 0.1$ and $y = 10$). Panel B: equilibrium X^L ($r = 1$ and $y = 2.5$). Panel C: equilibrium X_S^1 ($r = 1$ and $y = 0.6$). Panel D: equilibrium X_S^2 ($r = 0.255$ and $y = 3.9$).

2 Quantitative model calibration

Beyond the theoretical analysis of the model, the next step was to use experimental data in order to provide a quantitative estimation of the parameters, as close as possible to our experimental setup. We disposed of various datasets, consisting in temporal measurements of: *(i)* single-species S, *(ii)* competing species L and S with a lamB- mutation and *(iii)* competing species L and S without the mutation. All those experiments were made in monoxenic mice and the measurements techniques are described in the main text (Material & Methods). Profiting of the variety of these datasets, we analyzed the respective effects of each parameter into the model $(\mathcal{S})$ and decided to estimate the eight parameters in sequential blocks, as shown in Table A1. Six of them were estimated and two of them were hypothesized, as described below. We used classical estimation techniques (mainly non-linear least square regression) and all subsequent computations and figures were obtained with Matlab (The MathWorks, Inc.).

2.1 Estimation of growth parameters with single-species experiments

Parameters (r, k, d) directly command the dynamics of bacterial populations L and S. While d is global and does not depend on the bacterial strains, it is also assumed in the model that the maximal growth rate r and the carrying capacity k are the same for both populations. This assumption led us to estimate these parameters directly from single-species experiments, using a simple logistic differential equation including a dilution term:

$$\frac{dS}{dt} = r\left(1 - \frac{S}{k}\right)S - dS, \quad S(0) = S_0.$$

This equation can be solved analytically, giving the evolution of $S(t)$ in function of time:

$$S(t) = \frac{k(r-d)S_0 e^{(r-d)t}}{k(r-d) + rS_0(e^{(r-d)t} - 1)}.$$

Using datasets consisting in temporal evolutions of single-species S in mice, we used non-linear least square regression (`lsqnonlin` routine in Matlab), in logarithmic scale, to estimate r and k. Since global parameter d does not depend on the bacterial strains, we hypothesized $\bar{d} = 0.25$ (a value in line with other studies such as [1]) and estimated $\theta = (r, k)$ by minimizing:

$$\min_{\theta \in \Theta} \sum_{j=1}^{20} \sum_{i=1}^{10} \left(\log F_\theta\left(T_i^{data}\right) - \log S_{ij}^{data}\right)^2,$$

where $F_\theta(t)$ is given by the analytic solution of the logistic equation: $F_\theta(t) = \frac{k(r-d)S_0 e^{(r-d)t}}{k(r-d)+rS_0\left(e^{(r-d)t}-1\right)}$. For the optimization procedure we imposed $\Theta = [r_{min}, r_{max}] \times [k_{min}, k_{max}]$, with the following bounds:

$$r_{min} = 0, \qquad r_{max} = 2.5,$$
$$k_{min} = 1e9, \quad k_{max} = 1e11.$$

Using several runs with different initial conditions, taken on a hyper-rectangular regular grid centered on plausible parameter values, the procedure converged towards the following estimates: $\bar{r} = 1.1126$ and $\bar{k} = 4.62e9$. The result of the fit can be visualized in Figure A2.

In order to assess the variability of those values, we generated 500 simulated datasets using non-parametric bootstrap. Residues were computed in the original dataset: $R_{ij} = S_{ij}^{data} - \hat{S}_i$, where $\hat{S}$ is the geometric mean of time series S^{data}. Each additional dataset was then generated as the sum of $\hat{S}$ and residues taken uniformly randomly in the set $\{R_{ij}\}$. For each generated dataset, we launched the same minimization procedure, providing distributions for parameters r and k. Medians and 95% confidence intervals (computed with 2.5th and 97.5th percentiles) of those distributions are given below:

$$r_{median} = 1.1358, \quad r_{95\%} \in [1.0976, 1.1837],$$
$$k_{median} = 4.55e9, \quad k_{95\%} \in [4.26e9, 4.95e9].$$

Confidence intervals indicate a small variability (with relative precisions of 10^{-2} for r and 10^{-1} for k). Furthermore, the medians were close to $\bar{r}$ and $\bar{d}$, further suggesting a relatively high level of confidence in our estimates.

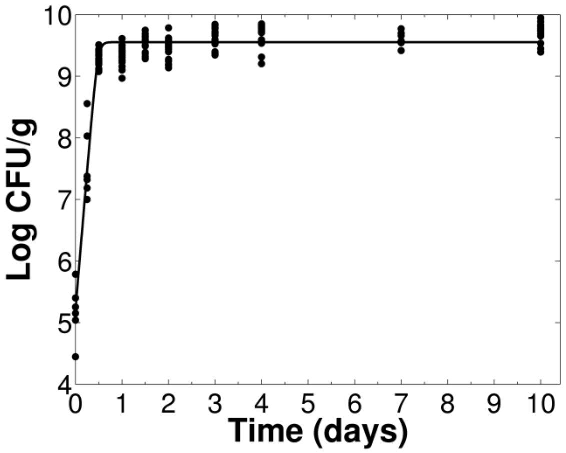

Figure A2. Estimation of growth parameters r, k. The dots represent temporal measurements of species S in mice (20 times series, each averaged on several mice) and the line represents the best fit of the logistic model F_θ using non-linear least square minimization ($\bar{r} = 1.1126$ and $\bar{k} = 4.62e9$).

2.2 Estimation of induction rate x and latency rate l with a lamB- model

In $(\mathcal{S})$, parameters x and l primarily command the dynamics of latent cells Q. In order to disentangle the estimation of x, l from the effects of phage-dependent parameters, we decided to use a lamB- version of Model $(\mathcal{S})$, in which $a = 0$ (*i.e.* the virus is unable to bind to bacteria). Since the variable $V(t)$ does not influence the other variables in this version, we only considered three ODE:

$$(\mathcal{S}') \quad \begin{aligned} \frac{dL}{dt} &= r\left(1 - \frac{N}{k}\right)L - xL - dL, L(0) = L_0, \\ \frac{dS}{dt} &= r\left(1 - \frac{N}{k}\right)S - dS, S(0) = S_0, \\ \frac{dQ}{dt} &= xL - lQ - dQ, Q(0) = Q_0. \end{aligned}$$

An analytical solution of this system is out of reach, implying the use of a numerical solver (`ode45` in Matlab). Only one dataset, taken from three mice over 4 days, was available for the estimation. We used the same technique as before, minimizing the sum of errors' squares, in logarithmic scale:

$$\min_{\theta\in\Theta} \sum_{i=1}^{7} \Big[\left(\log L_\theta\left(T_i^{data}\right) - \log L_i^{data}\right)^2 + \left(\log S_\theta\left(T_i^{data}\right) - \log S_i^{data}\right)^2 + \left(\log Q_\theta\left(T_i^{data}\right) - \log Q_i^{data}\right)^2\Big],$$

where $(L_\theta, S_\theta, Q_\theta)$ is the solution calculated by the numerical solver and $\theta = (x, l)$. Since the initial value Q_0 was missing, we assumed $Q_0 = 3.4\ 10^{-4} \times L_0$ (a value estimated from other datasets). As before, the optimization procedure was initialized with multiple starting points, and bounds were imposed on the parameters:

$$\begin{aligned} x_{min} &= 0, \quad r_{max} = 0.2, \\ l_{min} &= 0, \quad l_{max} = 1.5. \end{aligned}$$

We obtained the following estimates: $\bar{x} = 0.0159$ and $\bar{l} = 0.8315$ (see Figure A3). As before, we used a bootstrap technique to generate 500 simulated datasets and thus assess the variability of those estimates. Here, the residues were computed with respect to the estimated model: $R_{ij}^1 = L_i^{data} - L_{\bar{\theta}}\left(T_i^{data}\right)$, $R_{ij}^2 = S_i^{data} - S_{\bar{\theta}}\left(T_i^{data}\right)$, and $R_{ij}^3 = Q_i^{data} - Q_{\bar{\theta}}\left(T_i^{data}\right)$, with $\bar{\theta} = (\bar{r}, \bar{d})$. Simulated

datasets were then computed as the sum of $(L_{\bar{\theta}}, S_{\bar{\theta}}, Q_{\bar{\theta}})$ and residues taken uniformly randomly in the set $\{R_{ij}^1, R_{ij}^2, R_{ij}^3\}$. As expected, because of the small set of data used (one experiment with 3 mice), the distributions obtained for x and l are much broader than the ones obtained for r and k, indicating a lower confidence index.

$$x_{median} = 0.0159, \quad x_{95\%} \in [0.0063, 0.036],$$
$$l_{median} = 0.7685, \quad l_{95\%} \in [0,1.5].$$

In order to comfort the estimation of the induction rate x, we also calculated it from the relative fitness of L compared to S lamB lineages (see Material & Methods). The value found (0.017 ± 0.05) was very close, thus comforting the estimation.

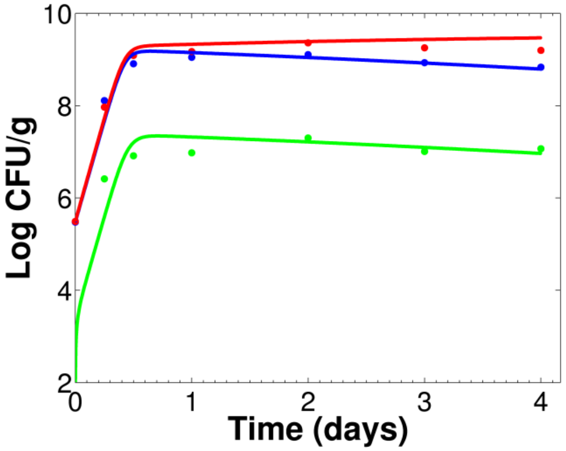

Figure A3. Estimation of parameters x, l. The dots represent the temporal measurements of species L, S, Q with a lamB- mutation in mouse (one dataset, averaged over three mice) and the lines represents the best fit of Model (S') using non-linear least square minimization ($\bar{x} = 0.0159$ and $\bar{l} = 0.8315$). Red: S, blue: L, green: Q.

2.3 Estimation of adsorption constant with free virus measurements

The next step consists in the estimation of phage-dependent parameters: the adsorption constant a and the burst size y. To do this, observe the formula of the steady state free virus in the original model $(\mathcal{S})$:

$$V^* = \frac{ylxk\left(1 - \frac{x+d}{r}\right)}{a(l+d+x)k\left(1 - \frac{x+d}{r}\right) + d(l+d)}.$$

With this formula, assuming V^* is given and using previously estimated values $\bar{r}, \bar{k}, \bar{d}, \bar{x}, \bar{l}$, a simple linear relation directly links the adsorption constant and the burst size: $\alpha \times y + \beta \times a + \gamma = 0$, with:

$$\alpha = \bar{l}\bar{x}\bar{k}\left(1 - \frac{\bar{x} + \bar{d}}{\bar{r}}\right),$$
$$\beta = -(\bar{l} + \bar{d} + \bar{x})\bar{k}\left(1 - \frac{\bar{x} + \bar{d}}{\bar{r}}\right)V^*,$$
$$\gamma = -\bar{d}(\bar{l} + \bar{d})V^*.$$

Therefore, we used L-S experimental datasets to estimate V^* and thus deduce a, y. To further disentangle a from y, we decided to assume $\bar{y} = 12.1$ (a value estimated from *in vitro* experiments, see Material & Methods) and compute $\bar{a}$ from the previous linear relationship. This assumption will need to be comforted in the future. Nevertheless, it is based on the following rationale: between the two

parameters, the adsorption constant is clearly dependent on the immediate environment of the cells, therefore it seems preferable to infer a from experimental data in the mouse gut. It remains to give an estimate of steady state viral population V^*. Since we know a mutation occurs in L-S experiments, this value needs to be chosen with care. In Figure A4, we show the mean values of V, taken at 6 different times (x-axis). On the y-axis, we show the corresponding value of estimated $\bar{a}$. We chose the value $\bar{a} = 2.62.10^{-9}$, which corresponds to the mean viral density V^* at 36 hours (last time where mutants are in minority). This value is in line with theoretical expectations. Indeed, adsorption rate is directly proportional to diffusion speed [2]. In a semi-solid environment such as the intestinal content, virus diffusion rate can diminishe between 10 and 1,000 fold [3-5]. Since we found $a = 3.10^{-7}$ in feces diluted in water, the estimated value in the gastrointestinal tract falls within expected values.

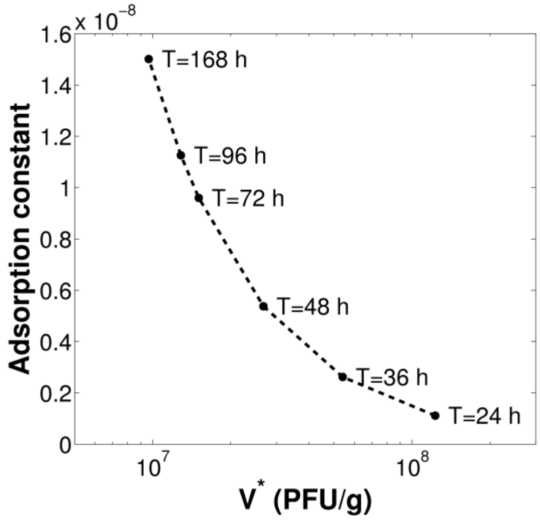

Figure A4. Estimation of the adsorption constant. The dots represent mean viral populations (x-axis) at different times (geometric mean of 8 different experiments) and the corresponding estimated value $\bar{a}$ (y-axis). The burst size is assumed constant $\bar{y} = 12.1$ (independently estimated from *in vitro* experiments).

2.4 Estimation of the probability of lysogenization g

Interestingly, parameter g does not intervene in any formula of the equilibrium, making it unique for that matter. Nevertheless, in the equilibrium, populations L^* and S^{l*} are only determined by their sum: $L^* + S^{l*} = k\left(1 - \frac{x+d}{r}\right)$. To verify if g has an impact on the relative positions of L and S^l, we computed the lysogen ratio $\rho(t) = L(t)/(S(t) + S^l(t))$ with Matlab, and used the value at time $t = 100h$ to approximate the asymptotic ratio: $\rho^* = L^*/(S^* + S^{l*})$, for the eight experimental L-S datasets. In each case, we initialized the simulation with initial measurements. We made this computation with previously estimated values for $\bar{r}, \bar{k}, \bar{d}, \bar{x}, \bar{l}, \bar{y}, \bar{a}$ and for different values of g in [0,1]. We clearly saw an impact of g, confirming this parameter directly controls the lysogen ratio ρ. Therefore, we used non-linear regression to minimize the following least square error:

$$\min_{g\in[0,1]} \sum_{j=1}^{8} \left(\log \rho_g^* - \log \rho_j^{data}(T)\right)^2.$$

In this formula, we had to consider different times T, to take into account the fact that experimentally, the steady state is only transient due to the mutation. As earlier, we tried different times and we obtained the minimal error at $T = 36h$, further comforting our earlier choice (see Figure A5). Moreover,

as can be seen in this figure each curve has a clear global minimum, allowing an unambiguous estimation. Finally, we obtained the following estimate: $\bar{g} = 0.186$.

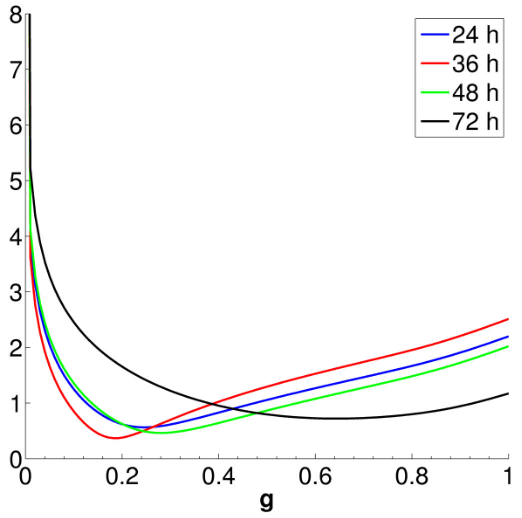

Figure A5. Estimation of the lysogenization rate. The curves represent the evolution with g of the error $\sum_{j=1}^{8}\left(\log \rho_g^* - \log \rho_j^{data}(T)\right)^2$. Each curve corresponds to different values of T (see upper right corner). In each case, the error clearly has a unique minimum, giving the best estimate of g. The global minimum is obtained for $T = 36h$, corresponding to $\bar{g} \approx 0.18$.

3 Sensitivity analysis

Let θ denote the vector $\theta = (d, r, k, x, l, y, a, g)$ and $\bar{\theta}$ be the vector of estimated values described previously. To analyze the sensitivity of the model with respect to θ and to assess the preciseness of our estimate $\bar{\theta}$, we briefly investigated the effect of a change in each individual parameter. More precisely, we computed the final value of lysogen ration $\rho^* = L^*/(S^* + S^{l*})$ (approximated at time $t = 100h$) with parameter $\theta^{(i)}$, where $\theta^{(i)}$ is $\bar{\theta}$ perturbed along dimension $i \in \{1, \cdots, 8\}$. The perturbations were chosen in certain ranges around plausible values, in normal or logarithmic scale according to the parameter. Table A2 below describes the chosen ranges of perturbation.

Table A2. Perturbation of parameters.

Parameter	Lower bound	Upper bound	Scale
d	0.1	0.4	Normal
r	0.7	1.5	Normal
k	3.5e9	1e10	Logarithmic
x	1e-4	0.1	Logarithmic
l	0.2	1.5	Normal
y	2	50	Normal
a	1e-10	1e-7	Logarithmic
g	1e-4	0.7	Logarithmic

The results are depicted in Figure A6. They show a clear effect of the lysogenization rate g, which seems to be the more sensitive parameter to this ratio. We also observe slight effects of the burst size y when it reaches low values (around 1) and of the induction rate x when it reaches high values around 10%. Otherwise, other parameters seem to have little to no effect to ratio ρ, at least in the tested ranges.

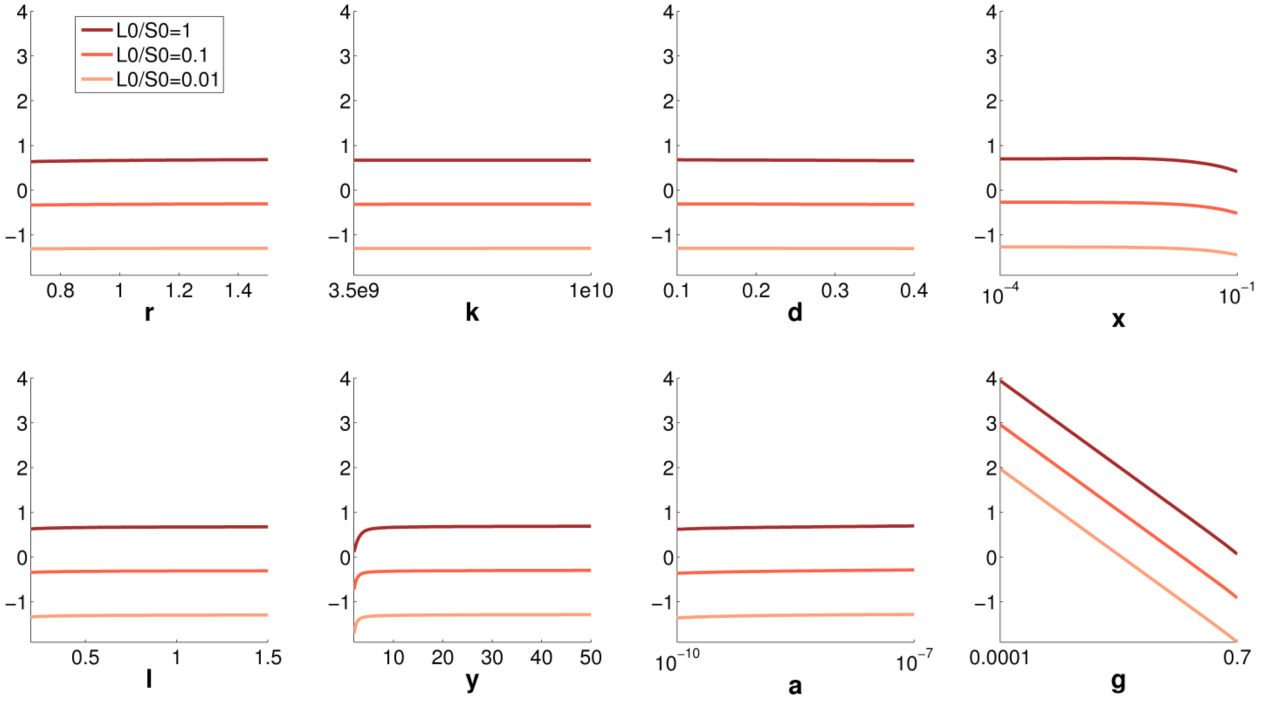

Figure A6. Sensitivity analysis. This figures show the evolution of final lysogen ratio $\log \rho^*$ when each parameter is perturbed individually within a range indicated in Table A2. The three colors correspond to three different initial ratios (see upper left panel).

4 References

1. Freter R, Brickner H, Fekete J, Vickerman MM, Carey KE (1983) Survival and implantation of Escherichia coli in the intestinal tract. Infect Immun 39: 686-703.
2. Delbruck M (1940) Adsorption of Bacteriophage under Various Physiological Conditions of the Host. J Gen Physiol 23: 631-642.
3. Lai SK, O'Hanlon DE, Harrold S, Man ST, Wang YY, et al. (2007) Rapid transport of large polymeric nanoparticles in fresh undiluted human mucus. Proc Natl Acad Sci U S A 104: 1482-1487.
4. Alvarez LJ, Thomen P, Makushok T, Chatenay D (2007) Propagation of fluorescent viruses in growing plaques. Biotechnol Bioeng 96: 615-621.
5. Olmsted SS, Padgett JL, Yudin AI, Whaley KJ, Moench TR, et al. (2001) Diffusion of macromolecules and virus-like particles in human cervical mucus. Biophys J 81: 1930-1937.
