# Supplementary material for: Carriage of λ Latent Virus Is Costly for Its Bacterial Host due to Frequent Reactivation in Monoxenic Mouse Intestine
Source: PLoS Genet. 2016 Feb 12;12(2):e1005861. doi: 10.1371/journal.pgen.1005861 (PMC4752277; doi:10.1371/journal.pgen.1005861)
Supplement: S1 Table — (DOCX) [file pgen.1005861.s006.docx]

**S1 Table: bacterial and phage strains**

| Strain | Genotype | Reference |
| --- | --- | --- |
| Bacterial strains | | |
| MG∆Y∆F | MG1655 ∆*fliC* ∆*ompF* | ([Giraud et al., 2008](#_ENREF_4)) |
| HME57 λ | W3110 Δ(*argF-lac*)U169 galK_tyr145UAG_ (λ *ble*) *pKD46* | this study and ([Datta et al., 2006](#_ENREF_2)) |
| MD19 | MG1655 *stfR::cat* | ([De Paepe et al., 2014](#_ENREF_3)) |
| MD5 | MG1655 ∆*fliC* ∆*ompF* *hsdR::KanR* | this study |
| MD6 | MG1655 ∆*fliC* ∆*ompF* *stfR::cat (*λ *ble)* | this study |
| MD47 | MG1655 ∆*fliC* ∆*ompF* *lamB*::KanR | this study |
| MD56 | MG1655 ∆*fliC* ∆*ompF lamB*::FRT *stfR::cat* (λ *ble*) | this study |
| MD74 | MG1655 ∆*fliC* ∆*ompF lamB*::FRT *stfR::cat* (λ *ble cI^ind-^*) | this study |
| JC10990 | AB1157 *recF332*::Tn3 *tnaA*::Tn10 | ([Blanar et al., 1984](#_ENREF_1)) |
| Phage strains | | |
| λ *ble* (WT) | Urλ *ble* | ([De Paepe et al., 2014](#_ENREF_3)) |
| λ *cI*^ind-^ | Urλ *ble* *cIA111T* | this study |

References

BLANAR, M. A., SANDLER, S. J., ARMENGOD, M. E., REAM, L. W. & CLARK, A. J. 1984. Molecular analysis of the recF gene of Escherichia coli. *Proc Natl Acad Sci U S A,* 81**,** 4622-6.

DATTA, S., COSTANTINO, N. & COURT, D. L. 2006. A set of recombineering plasmids for gram-negative bacteria. *Gene,* 379**,** 109-15.

DE PAEPE, M., HUTINET, G., SON, O., AMARIR-BOUHRAM, J., SCHBATH, S. & PETIT, M. A. 2014. Temperate phages acquire DNA from defective prophages by relaxed homologous recombination: the role of Rad52-like recombinases. *PLoS Genet,* 10**,** e1004181.

GIRAUD, A., AROUS, S., DE PAEPE, M., GABORIAU-ROUTHIAU, V., BAMBOU, J. C., RAKOTOBE, S., et al. 2008. Dissecting the genetic components of adaptation of Escherichia coli to the mouse gut. *PLoS Genet,* 4**,** e2.
